# Advances in understanding Norway spruce natural resistance to needle bladder rust infection: transcriptional and secondary metabolites profiling

**DOI:** 10.1186/s12864-022-08661-y

**Published:** 2022-06-13

**Authors:** Carlos Trujillo-Moya, Andrea Ganthaler, Wolfgang Stöggl, Erwann Arc, Ilse Kranner, Silvio Schueler, Reinhard Ertl, Ana Espinosa-Ruiz, Maria Ángeles Martínez-Godoy, Jan-Peter George, Stefan Mayr

**Affiliations:** 1 Department of Forest Growth, Silviculture & Genetics, Austrian Research Centre for Forests BFW, Seckendorff-Gudent-Weg 8, 1131 Vienna, Austria; 2 Department of Botany, University of Innsbruck, Sternwartestraße 15, 6020 Innsbruck, Austria; 3 University of Veterinary Medicine, VetCore Facility for Research, Veterinärplatz 1, 1210 Vienna, Austria; 4 Institute for Plant Molecular and Cell Biology (IBMCP), Consejo Superior de Investigaciones Científicas (CSIC) - Universidad Politécnica de Valencia (UPV), Valencia, Spain

**Keywords:** Conifer, Forest tree, Fungal infection, Host–pathogen-interaction, Phenolic compounds, PR Proteins, RNA sequencing, Transcriptomics, Background

## Abstract

**Background:**

Needle rust caused by the fungus *Chrysomyxa rhododendri* causes significant growth decline and increased mortality of young Norway spruce trees in subalpine forests. Extremely rare trees with enhanced resistance represent promising candidates for practice-oriented reproduction approaches. They also enable the investigation of tree molecular defence and resistance mechanisms against this fungal disease. Here, we combined RNA-Seq, RT-qPCR and secondary metabolite analyses during a period of 38 days following natural infection to investigate differences in constitutive and infection-induced defence between the resistant genotype PRA-R and three susceptible genotypes.

**Results:**

Gene expression and secondary metabolites significantly differed among genotypes from day 7 on and revealed already known, but also novel candidate genes involved in spruce molecular defence against this pathogen. Several key genes related to (here and previously identified) spruce defence pathways to needle rust were differentially expressed in PRA-R compared to susceptible genotypes, both constitutively (in non-symptomatic needles) and infection-induced (in symptomatic needles). These genes encoded both new and well-known antifungal proteins such as endochitinases and chitinases. Specific genetic characteristics concurred with varying phenolic, terpene, and hormone needle contents in the resistant genotype, among them higher accumulation of several flavonoids (mainly kaempferol and taxifolin), stilbenes, geranyl acetone, α-ionone, abscisic acid and salicylic acid.

**Conclusions:**

Combined transcriptional and metabolic profiling of the Norway spruce defence response to infection by *C. rhododendri* in adult trees under subalpine conditions confirmed the results previously gained on artificially infected young clones in the greenhouse, both regarding timing and development of infection, and providing new insights into genes and metabolic pathways involved. The comparison of genotypes with different degrees of susceptibility proved that several of the identified key genes are differently regulated in PRA-R, and that the resistant genotype combines a strong constitutive defence with an induced response in infected symptomatic needles following fungal invasion. Genetic and metabolic differences between the resistant and susceptible genotypes indicated a more effective hypersensitive response (HR) in needles of PRA-R that prevents penetration and spread of the rust fungus and leads to a lower proportion of symptomatic needles as well as reduced symptom development on the few affected needles.

**Supplementary Information:**

The online version contains supplementary material available at 10.1186/s12864-022-08661-y.

## Background

Spruce forests are the most widespread forest communities in the subalpine belt up to the treeline in Central Europe [1]. There, Norway spruce (*Picea abies* L. Karst) is one of the naturally most abundant conifers, being also promoted by forestry for its growing properties, effective natural rejuvenation, high productivity, and high-quality wood [2]. High elevation spruce forests provide many economical and socio-ecological ecosystem services, such as wood production, soil protection, water retention, and recreational use [3]. Of particular importance is their protective role for infrastructure and settlements against avalanches, rock falls and landslides. Biotic stress factors, including herbivorous insects, bacteria, viruses or fungi, constitute a major threat for these forests, negatively affecting tree survival, growth, and vitality [2]. Global climate change is expected to further aggravate the problem, as altered climatic conditions may cause more favourable conditions for pathogen survival and reproduction, and at the same time impair tree fitness and defence responses [4].

One of the most prevalent needle diseases in subalpine spruce forests is caused by the indigenous rust fungus *Chrysomyxa rhododendri* [5, 6]. This biotrophic parasite undergoes a complex life cycle with a host shift between rhododendrons (*Rhododendron* spp.) and Norway spruce and its occurrence is therefore restricted to high elevation areas, where both hosts co-occur. The infection of Norway spruce occurs in spring. Ripening spore stocks on rhododendron plants release basidiospores, which are wind-dispersed and enter the newly sprouting, current-year needles of spruce trees with a germination tube [7]. Continuous growth of the mycelium within the needles causes progressive yellowing of the foliage during summer and enables the fungus to form aeciospore stocks, where after dicaryotization and mitotic splitting aeciospores are released [6]. As fungal growth is restricted to individual current-year needles and infected needles are shed in autumn, each spring a new infection cycle starts and infection intensities as well as subsequent defoliation can strongly vary between individual years [7, 8].

Accordingly, moderate infection intensities in single years have little impact on the whole-tree photosynthetic activity and stem growth (losses can be compensated by healthy current-year and older needles [9]), but severe defoliation caused by repeated infections negatively affects multiple tree anatomical, morphological, and physiological traits. This leads to reduced radial and height growth or, especially in young saplings, even to cripple growth and dieback [6, 10, 11]. As a result, increasing difficulties in both natural regeneration and afforestation of subalpine spruce forests arise. Thus, there is a strong need to enhance our understanding of the genetic and biochemical response mechanisms of Norway spruce to needle rust infection and the natural variation in disease resistance.

Key principles of Norway spruce defence mechanisms against *C. rhododendri* were recently described based on a greenhouse-controlled infection experiment performed with genetically identical clone cuttings [12]. These in-depth molecular analyses revealed that infection induced-defence responses occur mainly in attacked symptomatic needles (local response) and trigger a hypersensitive reaction (HR) that prevents the fungus from spreading within the needle. This process includes the production of a complex combination of proteins and secondary compounds, such as endochitinases and flavonoids that helps isolate the fungus and halt its growth. This reaction was shown to occur mainly between 9–21 days post infection (dpi) and to persist at least until the symptoms of infection are visible (39 dpi; [12]). Moreover, it was shown that upon infection, plant-pathogen interaction, MAPK signalling, and plant hormone signal transduction pathways were differentially regulated, and both the primary and secondary metabolism of infected plants were highly affected. Several transcripts differentially expressed in infected needles were related to the biosynthesis of phenolic compounds and terpenes. The antifungal activity of these metabolites has been extensively described and is considered to play a central role in conifer response to fungal pathogens [13–16].

Within spruce populations, variation in susceptibility to *C.* *rhododendri* can be observed and susceptibility is presumable a quantitative trait with large phenotypic variation [11, 17, 18]. Naturally occurring trees with remarkably enhanced resistance are extremely rare [13], but represent ideal study objects to elucidate response mechanisms of spruce to fungal needle infection, and to identify genetic and biochemical markers for this observed resistance [19]. At the same time, they are promising candidates for practice-oriented approaches to reproduce resistant trees. Most genetic and biochemical studies on plant resistance to fungal pathogens have concentrated on important model organisms and crops, mainly in controlled greenhouse experiments (e.g. [20, 21]). Resistance mechanisms of forest trees, and especially of high-elevation trees, received comparably little attention and mainly with respect to wood and root pathogens (e.g., [22, 23]) and qualitative resistance (e.g. [24]). Thus, analyses of functional aspects of tree resistance to foliar pathogens like *C.* *rhododendri* and measurements of adult trees in the field will help providing new insights relevant also for other conifer foliar diseases and related practical aspects.

**Fig. 1:**
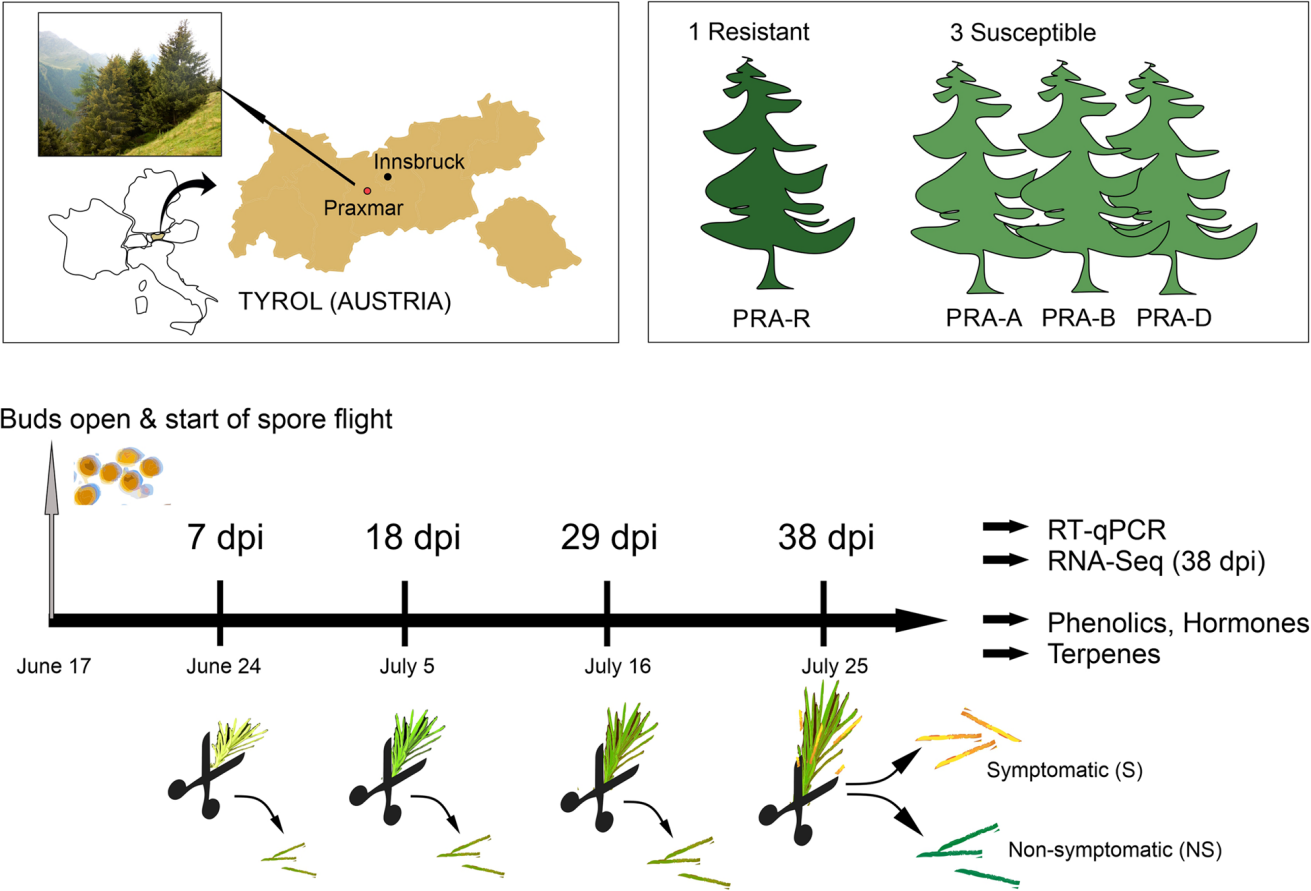
Study design and sampling procedure. Needles were sampled from one tree with enhanced resistance and three susceptible adult Norway spruce trees growing next to each other in an area with naturally high *C.* *rhododendri* infection intensities (Praxmar in Tyrol, Austria). On each sampling date and for each tree, three current-year shoots from different parts of the crown were cut, the needles of each divided into four cryo vials (for RNA-Seq, phenolics, terpenes, and backup, respectively) and immediately stored at -80° C. Note that on day 38 infection symptoms were clearly visible and needles were separated into symptomatic (S) and non-symptomatic (NS) ones

In the present study, we combined genetic and secondary metabolite analyses to better understand the mechanisms underlying the enhanced resistance of adult Norway spruce trees against *C.* *rhododendri* under field conditions at the subalpine treeline, taking advantage of a previously identified genotype with known enhanced resistance (PRA-R) that was compared to highly susceptible control trees (Fig. 1). PRA-R was recognized already years ago as one of the genotypes with most distinct resistance to needle rust and was thus subject to several studies [9, 11, 13]. We chose to conduct repeated analyses within a period of 38 days following the start of infection including symptomatic (S) and non-symptomatic (NS) needles to identify whether the resistant clone PRA-R showed constitutive defence (always present in the plant) and/or induced defence (activated when infection occurs) on the molecular level, regarding involved genes and metabolic pathways and/or timing of their activation. Several investigations on biotrophic fungi in conifers revealed a strong induced response following the recognition of specific effectors [24, 25], but constitutive defence can represent a first line of resistance and both can be combined to an efficient defence strategy [15, 26]. NS needles of infected trees were previously shown to correspond to healthy needles of unaffected trees [12] and thus can reveal the constitutive tree defence and at the same time can be used as reference to study the induced defence in symptomatic needles.

We hypothesized that (1) an induced and/or constitutive defence mechanisms is responsible for plant performance when challenged with the pathogen, (2) differences in both constitutive and inducible defence contribute to the higher resistance of genotype PRA-R, and (3) differential gene expression concurs with specific changes in phenolic compounds, terpenes, and plant hormones, which have a key role in plant defence to biotic stress factors. Once symptoms of infection were clearly visible (38 dpi), RNA-Seq was used to compare induced and constitutive defence mechanisms (on S and NS needles, respectively) between genotypes, and to compare the new data with results previously obtained under controlled greenhouse conditions [12]. Furthermore, samples from all time points were analysed by RT-qPCR (using a set of 30 selected genes), GC–MS (terpenes) and LC–MS/MS (flavonoids, stilbenes, shikimic acid, and hormones) to explore differences in defence mechanisms at early to late stages of infection including both gene expression and metabolite level.

## Results

### Symptom development

**Fig. 2:**
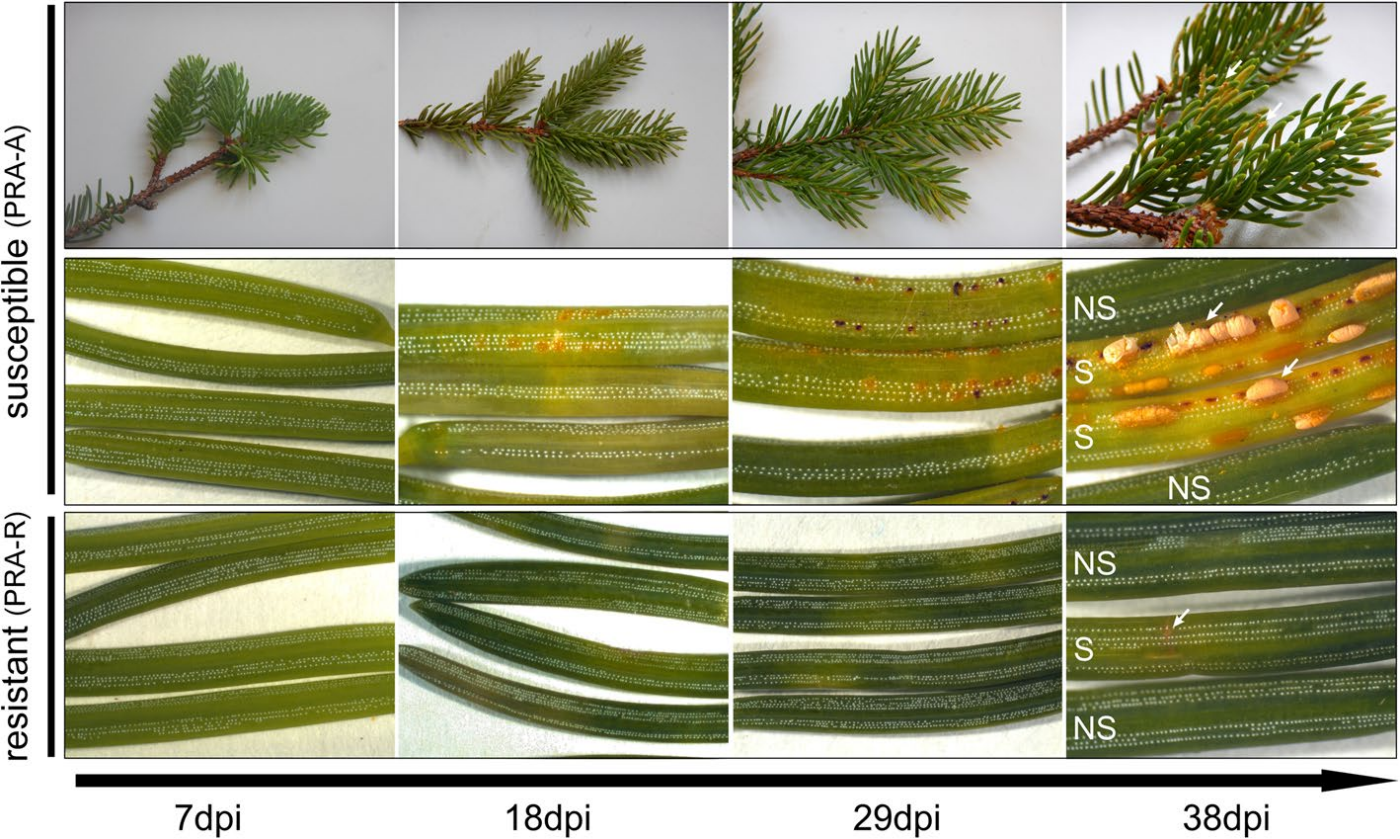
Symptom development of *C. rhododendri-*infections over time of PRA-A (representative of susceptible genotypes) and of the resistant genotype PRA-R needles. At 18 dpi, first symptoms of infection were detectable under the microscope, i.e., small blisters on the needle surface become visible on symptomatic (S) needles while other needles remained non-symptomatic (NS). At 38 dpi, several current-year needles of susceptible trees showed a characteristic yellow discoloration and first aeciospore stocks were formed, while PRA-R was only slightly affected*,* with tiny yellow spots on S needles (arrows indicate symptoms)

The first infection symptoms (small blisters on the needle surface) appeared from 18 dpi on, whereas the clear yellow discoloration was only visible about ten days later (Fig. 2). This time frame corresponded to the infection progress described under controlled conditions [12]. In susceptible trees, 32 to 58% of current-year needles developed clear infection symptoms with extensive needle discoloration and formation of large spore stocks (Fig. 2; Additional file 1: Table S1). In contrast, the resistant genotype (PRA-R) was only marginally affected with tiny yellow–brown spots on about 3% of current-year needles, showing no large-scale discoloration and no spore stocks, thus clearly differing from S needles of susceptible trees.

### Thirty-eight dpi RNA-Seq analysis

A total of 21.14 million single-end reads (median across samples) were obtained from cDNA sequencing (Illumina NextSeq 500) of the libraries (QuantSeq 3' mRNA-Seq FWD) generated from needles collected at 38 dpi. The number of input reads (after quality control trimming) varied between 17.26 million and 22.74 million depending on treatment and genotype (Additional file 2: Table S2). The number of reads that uniquely aligned to a location in the Norway spruce reference genome varied between 6.95 million and 17.05 million (from 36 to 78% of the total reads). Average input read length varied slightly between 72 and 74 nt. Correlation between the three technical replicates for each treatment and genotype combination was high in all cases, suggesting that cDNA sequencing yielded reliable results for downstream analyses (Additional file 3: Figure S1).

**Fig. 3:**
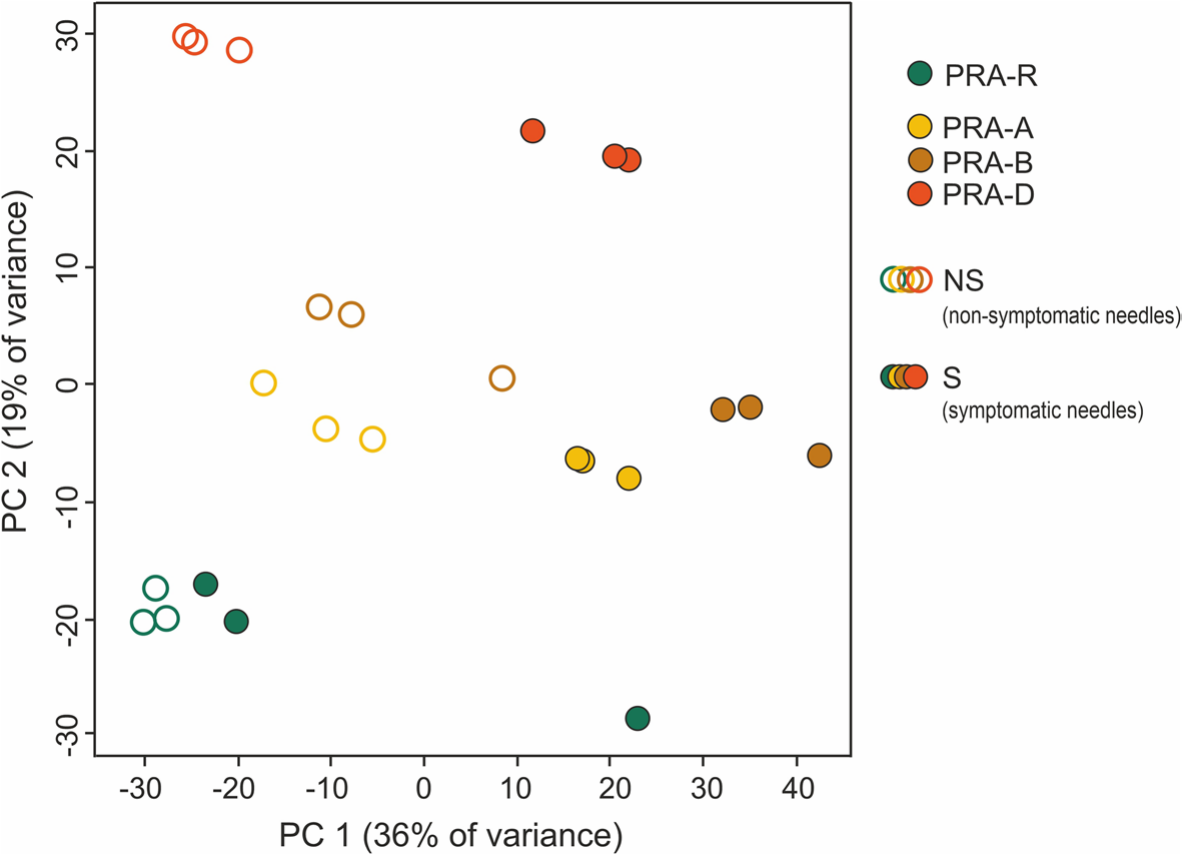
Principal component analysis (PCA) applied to differentially expressed genes at 38 dpi. Note the clear separation of individual genotypes and of S and NS needles, except for PRA-R

Principal component analysis (PCA) applied to the gene expression profiles revealed large differences between the genotypes, which were clearly separated by the second principal component (PC2) with the resistant genotype PRA-R on one end of the spectrum (Fig. 3). In addition, in susceptible genotypes, S and NS needles clustered separately along the first principal component (PC1) indicative of difference in their gene expression profiles, whereas PRA-R S and NS needles could not be clearly differentiated.

#### Induced defence

**Fig. 4:**
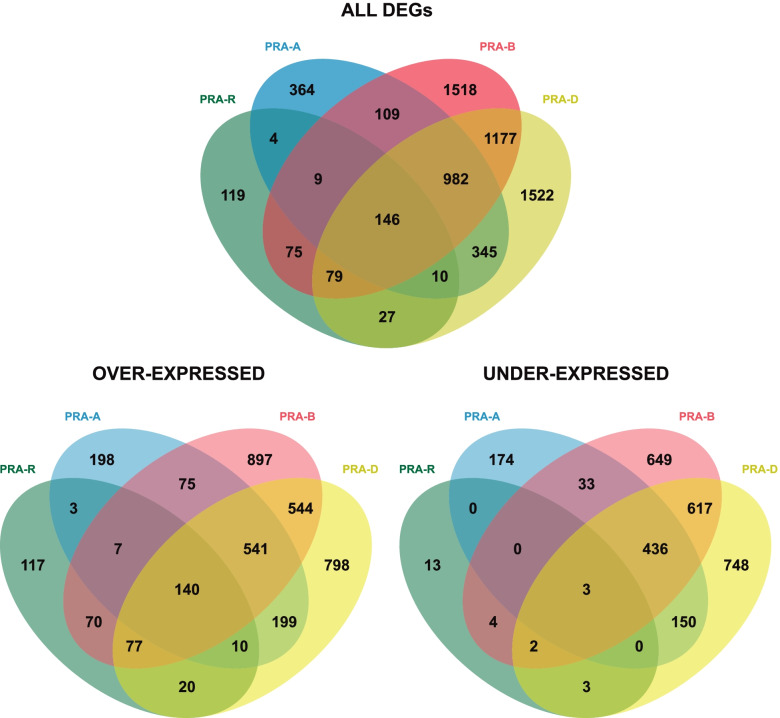
Induced differences in gene expression between the symptomatic and non-symptomatic needles for each genotype. Venn diagrams illustrating the overlap between differentially expressed genes for each genotype in S needles compared to NS needles at 38 dpi

The induction of defence responses was assessed by comparing the gene expression in S compared to NS needles of the same tree. Whereas 1969, 4095 and 4288 transcripts were differentially expressed in the susceptible genotypes PRA-A, PRA-B and PRA-D, respectively, only 469 differentially expressed genes (DEGs) were found in PRA-R (Fig. 4; Additional file 4: Table S3 A-D). Only 146 DEGs were shared by all individuals, whereas 982 genes were differentially expressed in all susceptible genotypes. In addition, in susceptible genotypes 57% of DEGs were over-expressed compared to 95% in PRA-R (Additional file 5: Figure S2).

Significantly enriched Gene Ontology (GO) terms were identified for all analysed genotypes but particularly for susceptible genotypes in over-expressed (38 terms on average) and under-expressed transcripts (36 terms on average) (for the detailed list see Additional file 6: Figure S3). Among the over-expressed transcripts, the most enriched terms for biological processes were related to: response to stress, cellular processes, metabolic processes, catabolic processes, transport, response to biotic and abiotic stimulus, signal transduction. The most enriched terms for cellular components were membrane, cytosol, plasma membrane and intracellular aspects, while for molecular function catalytic activity, hydrolase activity, transferase activity, nucleotide binding and kinase activity. For under-expressed transcripts, GO terms such as photosynthesis and subsequently thylakoid/plastid as well as generation of precursor metabolites and energy were enriched. The resistant genotype PRA-R with only few DEGs showed similar enriched GO terms as found for susceptible genotypes in both over-expressed (ten terms) and under-expressed (five terms) transcript sets.

The Kyoto Encyclopedia of Genes and Genomes (KEGG) database was also used to categorize gene function [27–29]. On average, 34% of DEGs could be assigned to enzymes involved in known biological pathways (Additional file 7: Table S4). Due to the complexity of the Norway spruce genome (large gene family expansions on defence related pathways; [30]), several genes were assigned to the same enzymes (Additional file 8: Table S5, Additional file 9: Table S6). In susceptible genotypes, several genes encoding enzymes involved in the MAPK signalling, plant hormone signal transduction and the plant-pathogen interaction pathways (for which several under-expressed genes were also found), and in the starch and sucrose metabolism were over-expressed, whereas many genes coding for enzymes involved in photosynthesis, porphyrin and chlorophyll metabolism, carbon fixation and glyoxylate and dicarboxylate metabolism were under-expressed. Interestingly, many genes related to the translation machinery were under-expressed (ribosomal proteins), whereas those associated to protein processing in the endoplasmic reticulum remained stable. The low number of DEGs found for the resistant genotype PRA-R made a KEGG analysis uninformative in this particular case.

DEGs distribution across pathways (pathway fingerprint) was compared with data from the previous greenhouse controlled infection experiment performed on the clone ASS-7 [12]. The heat map (Additional file 10: Figure S4) showed high similarity between the response to infection of susceptible adult trees in the field (38 dpi) and of young and artificially infected clones of the ASS-7 genotype (analysed at 39 dpi), both when considering the comparison of ASS-7 symptomatic needles (S) to healthy needles from non-infected control plants (S-C) and to NS needles of the same (infected) plant (S-NS). This congruence also applied to ten KEGG pathways that were previously identified to be involved in the defence [12], including for example plant-pathogen interaction, MAPK signalling, and plant hormone signal transduction (Additional file 11: Figure S5).

#### Constitutive defence

**Fig. 5:**
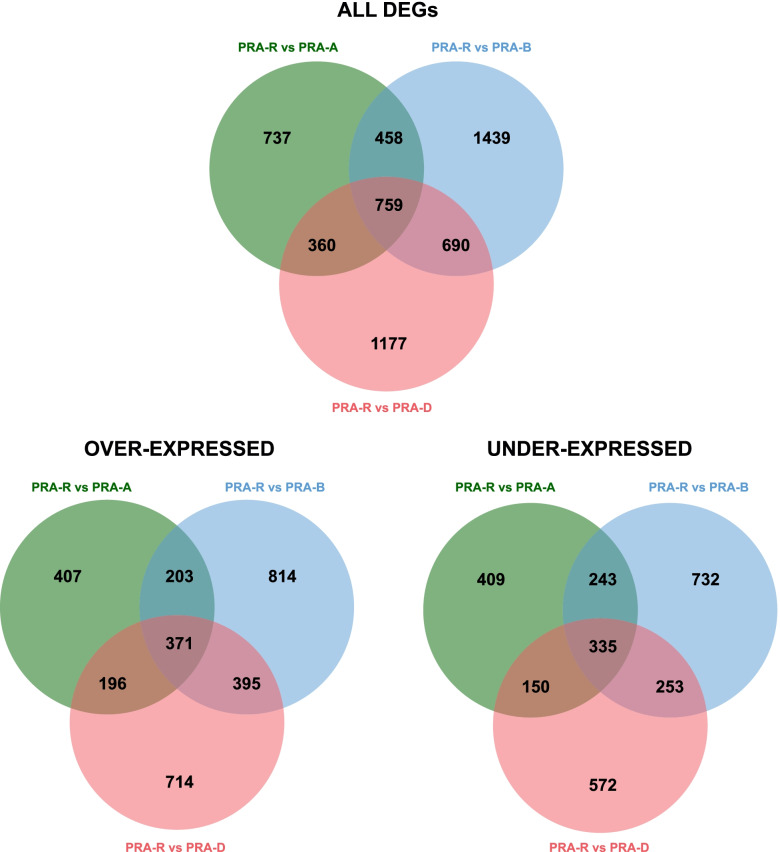
Constitutive differences in gene expression between the resistant and susceptible genotypes. Venn diagrams illustrating the overlap between genes differentially expressed when PRA-R NS needles were compared with PRA-A, PRA-B, and PRA-D NS needles at 38 dpi

Gene expression in NS (uninfected and healthy) needles was then compared between the resistant and each of the susceptible genotypes to identify specific constitutive defence mechanisms in PRA-R. Overall, 2314, 3346 and 2986 genes were differentially expressed compared to PRA-A, PRA-B and PRA-D, respectively (Fig. 5; Additional file 4: Table S3 E–G; Additional file 5: Figure S2). On average, 53% of DEGs were over-expressed and 38% (32–43%) were unique. In turn, 706 transcripts were shared by all comparisons (371 over-expressed and 335 under-expressed), representing 25% (21–31%) of all DEGs (Additional file 12: Table S7).

Significantly enriched GO terms were found for all comparisons (Additional file 13: Figure S6) in both, over- (19 terms on average) and under-expressed gene sets (21 terms on average). Among the over-expressed genes in PRA-R, the most enriched terms for biological processes were related to metabolic process, cellular process, response to stress, and response to biotic, abiotic and endogenous stimulus. Catalytic, transferase and kinase activities were the most enriched terms for molecular function. For under-expressed genes, the most pronounced differences were observed for lipid metabolism, metabolism, biosynthesis and secondary metabolism. Similar results were obtained when the analysis focused only on the 706 shared DEGs (Additional file 13: Figure S6).

With regard to biochemical pathways, on average, 28% of DEGs could be assigned to enzymes (Additional file 14: Table S8). DEGs distribution across pathways (pathway fingerprint) was similar for all pairwise comparisons of PRA-R and susceptible genotypes (Additional file 15: Figure S7 A). Focusing on the subsets of shared over- and under-expressed DEGs (Additional file 15: Figure S7 B), we found that several enzyme-encoding genes previously identified as highly relevant in the spruce response to needle rust infection [12] were differentially expressed in the resistant tree PRA-R (Additional file 15: Figure S7 B; Additional file 16: Table S9; Additional file 17: Table S10).

### RT-qPCR of selected DEGs

**Fig. 6:**
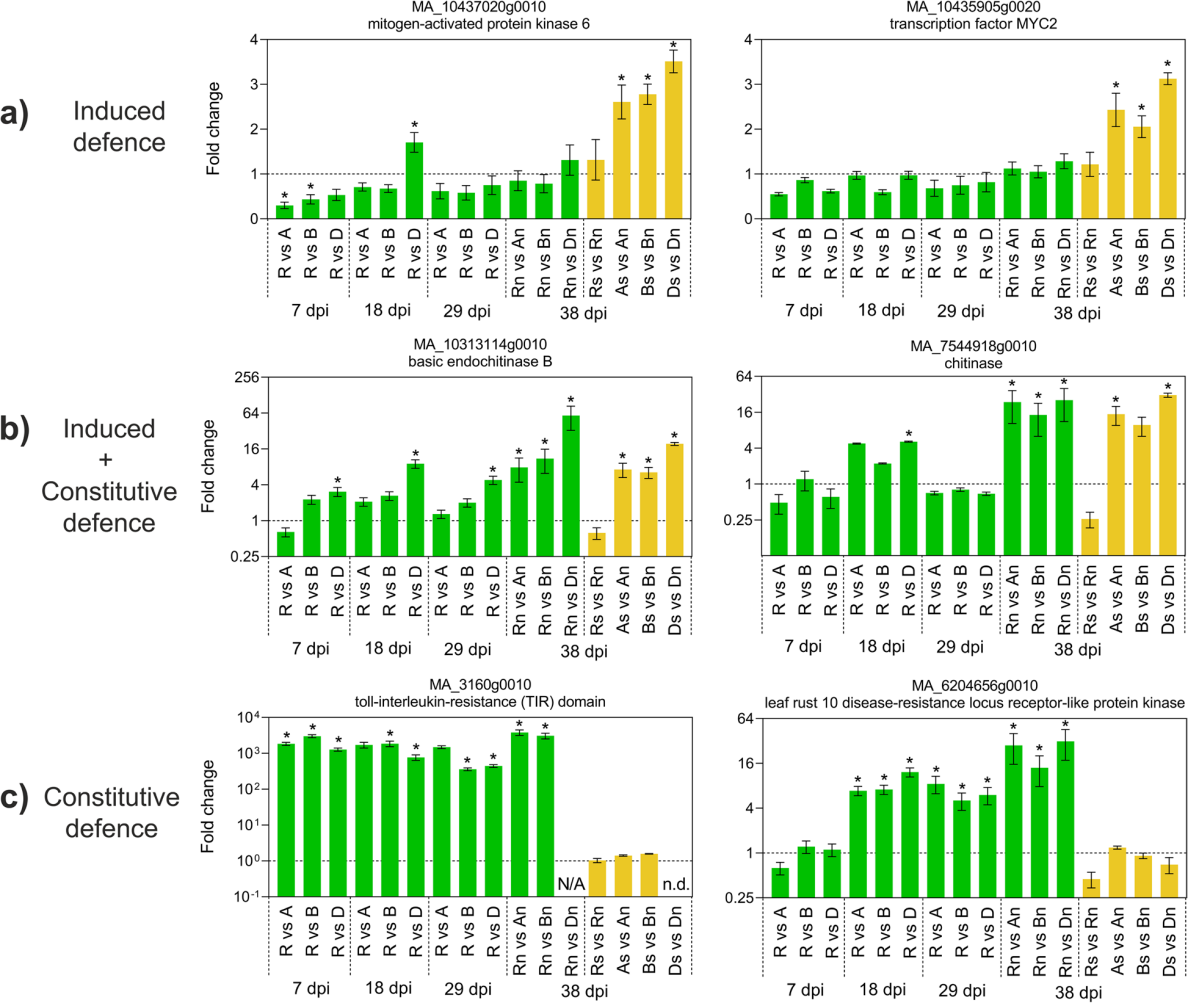
RT-qPCR results for 6 selected differentially expressed genes illustrating three main types of expression patterns indicative of: **a** induced defence, **b** induced and constitutive defence, **c** constitutive defence. All displayed genes belong to the MAPK signalling and plant hormone signal transduction pathways (for the whole set of 30 genes explored by RT-qPCR see Additional file 18: Figure S8). Relative expression changes (mean fold change ± SE, *n* = 3) were calculated for resistant (R) vs. susceptible genotypes (A, B, D) and symptomatic (S) vs. non-symptomatic (NS) needles at 7, 18, 29, and 38 dpi. * *p* < 0.05 (Welch’s t-test). N/A: fold changes could not be calculated as the respective transcript was not detected in one of the comparison partners; n.d.: transcript was not detected in both comparison partners

A set of 30 genes was selected for RT-qPCR to validate their induction in S needles at 38 dpi and to explore their differential expression between genotypes during the entire infection process including 7, 18, and 29 dpi. First, 15 genes previously identified by [12] as part of the induced defence response (Fig. 6 A; Additional file 18: Figure S8 A “orange delimited”) were confirmed in all susceptible genotypes. The highest over-expressions in S needles were found for the peroxidase gene MA_66201g0010 (49-fold) and genes encoding well-known antifungal proteins [31–33] including two basic endochitinases B (CHIB, MA_8921185g0010 and MA_10313114g0010), a defensin (MA_1489g0010) and a class IV chitinase (CHI4; MA_10427514g0010) (Additional file 18: Figure S8, Additional file 19: Table S11). Moreover, the basic endochitinase B gene (MA_10313114g0010) also showed a higher expression in NS needles of PRA-R in comparison to all susceptible genotypes (17-fold; Fig. 6 B), while the opposite pattern was found for the chalcone synthase gene MA_5735g0010 (at 38 and 29 dpi), similar to the pathogenesis-related protein 1 gene (MA_53673g0010) and a cinnamyl-alcohol dehydrogenase gene (MA_10432110g0010). Finally, mitogen-activated protein kinase 6 (MA_10437020G0010), peroxidase, class IV chitinase and again, chalcone synthase genes showed a lower expression in the resistant genotype at early stages (7 dpi) of infection.

Second, genes coding for two additional proteins, a salt stress response/antifungal protein (MA_10430127g0010) and a chitinase (MA_7544918g0010) were selected for validation due to their opposite performance in susceptible (over-expression) and resistant genotypes (under-expression) S needles (Fig. 4; Additional file 18: Figure S8 A). RT-qPCR confirmed these patterns and additionally revealed higher expression of the antifungal protein gene in the resistant genotype (PRA-R) NS needles (Fig. 6 B), even from 29 dpi on.

Third, we investigated genes highly relevant for flavonoid biosynthesis (flavonol synthase; MA_4711g0010), terpenoid backbone biosynthesis (farnesyl diphosphate synthase; MA_175884g0010) and diterpenoid biosynthesis (ent-copalyl diphosphate synthase; MA_9401581g0010) that were differentially expressed in all susceptible genotypes but not in PRA-R (Additional file 18: Figure S8 A). RNA-Seq results were confirmed for all over-expressed genes except the under-expressed flavonol synthase gene that actually was lower at 7 dpi in PRA-R.

Fourth, a gene coding for a carotenoid cleavage dioxygenase (CCD; MA_10435932g0010) that has been suggested to be involved in the production of apocarotenoids [34], was explored together with genes encoding the flavonoid 3´,5´-hydroxylase (F3´5´H; MA_10430573g0010) and flavanone-3-hydroxylase (F3H), which were reported to play an important role in biosynthesis of phenolics in spruce in response to the bark beetle-associated fungus *Endoconidiophora polonica* [35, 36]. RT-qPCR showed higher expression of this CCD gene in the resistant genotype at 29 dpi, but lower expression at early stages of the infection whereas the flavanone-3-hydroxylase gene was the only over-expressed in the resistant genotype at 18 dpi.

**Fig. 7:**
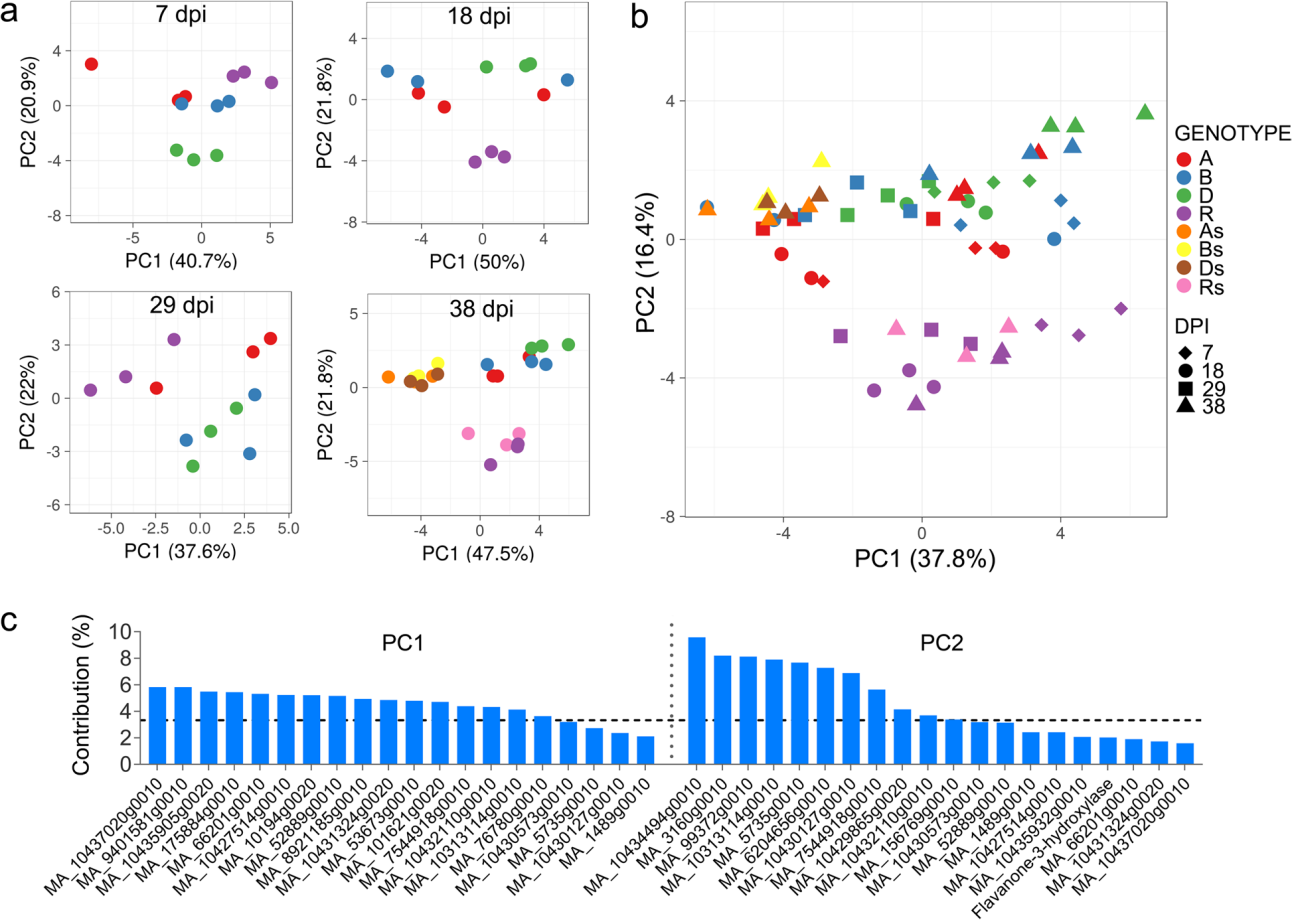
Principal component analysis (PCA) of RT-qPCR analysed transcripts. **a** Separate PCA plots for each analysed time point and **b** PCA plot including all samples. Note the clear separation of resistant and susceptible genotypes and of S and NS needles (animated version: Additional file 20: animation S1). At 38 dpi, S needles (As, Bs, Ds, Rs) are represented by an additional colour in contrast to the NS needles (A, B, D, R). **c** Contribution of individual transcripts to principal components 1 and 2 for PCA plot including all samples. Top 20 most contributing transcripts are shown for each component. The dashed horizontal line indicates the expected average contribution

Fifth, seven putatively defence-related genes, selected from the 706 shared transcripts (NS needles, PRA-R versus PRA-A/B/D) were validated (Additional file 18: Figure S8 B; Additional file 12: Table S7). The resistant genotype showed higher expression of toll-interleukin-resistance TIR domain (MA_3160g0010), Leaf Rust 10 disease-resistance locus (MA_6204656g0010) and cytochrome P450 (MA_99372g0010) genes at most time points (Fig. 6 C), while the sesquiterpene synthase gene (MA_10434494g0010) was consistently under-expressed. Differences between genotypes and between PRA-R and susceptible genotypes at early time points are well-visible in the PCA (performed using normalized expression values (ΔCq) of all 30 genes obtained by RT-qPCR; Fig. 7; for animated version Additional file 20: Animation S1).

### Plant secondary metabolites

#### Volatile terpenes

**Fig. 8:**
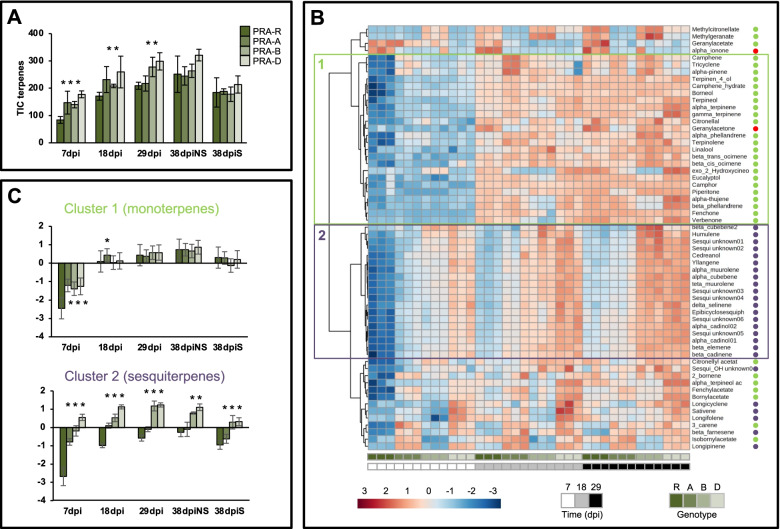
Terpene accumulation pattern after infection. **a** Total terpenes in the genotypes PRA-R, PRA-A, PRA-B and PRA-D at different time points. Note that PRA-R accumulated less terpenes at earlier time points, and that this difference decreased with needle age. **b** Hierarchical cluster analysis of terpene content from 7 to 29 dpi. Colours in the heat map represent the log2 ratio, according to the scale shown at the bottom. Green dots designate monoterpenes, red dots apocarotenoids and purple dots sesquiterpenes. **c** Terpene abundance, represented as log2 ratio referred to the reference mix, for metabolites belonging to groups 1 and 2 (shown in panel B). Note that sesquiterpenes are less abundant in PRA-R at all time points. Asterisks represent statistically significant difference (Ttest, *p*value < 0.05) between each genotype and PRA-R total terpene content

A total of 60 terpenes were identified: two apocarotenoids, 33 monoterpenes and 25 sesquiterpenes (Additional file 21: Table S12). Total terpene contents in all four genotypes were found to increase with time until 29 dpi (likely a general accumulation pattern during needle development) [37], and at 38 dpi became stable in NS needles, whereas they decreased in S needles (Fig. 8 A). PCA revealed a time-dependent differential profile for the four genotypes (Additional file 22: Figure S9). At 7 dpi, the PCA score plot clearly separated PRA-R from susceptible genotypes along PC1 explaining 58.3% of the total variability. At 18 and 29 dpi, PRA-R and PRA-A were closer and at 38 dpi they were no longer separated along any principal component.

A hierarchical cluster analysis for 7, 18, and 29 dpi (Fig. 8 B) confirmed that PRA-R contained lower amounts of most metabolites than the susceptible genotypes at 7 dpi, but over time the genotypes PRA-R and PRA-A became more similar and distinct from the genotypes PRA-B and PRA-D. The terpenes grouped in four main clusters, whereby clusters 1 and 2 represented more than 70% of total terpenes identified and were composed mainly of mono and sesquiterpenes, respectively. Clustered metabolites showed a highly synchronized accumulation pattern (Fig. 8 B), but each cluster showed a different development over time (Fig. 8 C). While monoterpenes were less abundant in PRA-R only at 7 dpi and accumulated to similar levels in all genotypes at later times, sesquiterpenes remained lower in PRA-R at all time points tested (Fig. 8 C). Interestingly, 3-carene (monoterpene) and β-farnesene (sesquiterpene) were consistently less abundant whereas geranyl acetone, α-ionone (*bona fide* apocarotenoids according to [38]) and geranyl acetate were consistently higher in PRA-R at all time points (Additional file 23: Figure S10). The remaining clusters were composed of small groups of metabolites that varied across the four genotypes; eight out of 17 were methyl and ethyl esters.

**Fig. 9:**
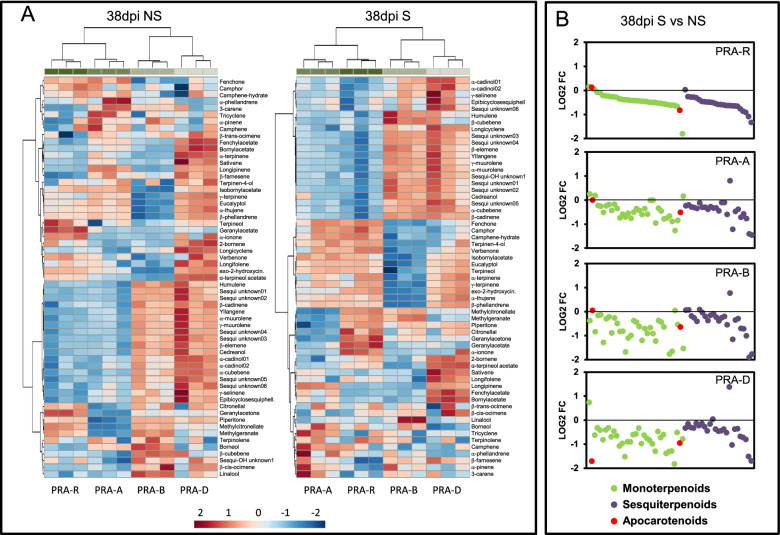
Terpene accumulation pattern at 38 dpi in symptomatic (S) and non-symptomatic (NS) needles. **a** Hierarchical cluster analysis of terpene contents at 38 dpi, in S and NS needles. Colours represent the log2 ratio, according to the scale shown at the bottom Note that the genotypes PRA-R and PRA-A show a very similar terpene accumulation pattern. **b** Terpene abundance, represented as log2fold change of S vs. NS needles for each genotype. Note the general terpene decrease in S needles

At 38 dpi, terpene contents were very similar in the genotypes PRA-R and PRA-A for both S and NS needles (Fig. 9 A), in agreement with the clustering on the PCA plot (Additional file 22: Figure S9). The most obvious difference between S and NS needles was the generally lower terpene content in S needles (Fig. 9 B). Compared to the susceptible genotypes, geranyl acetate was higher in both S and NS needles of PRA-R, and terpineol and geranyl acetone in NS needles and α-ionone in S needles.

#### Phenolic compounds

**Fig. 10:**
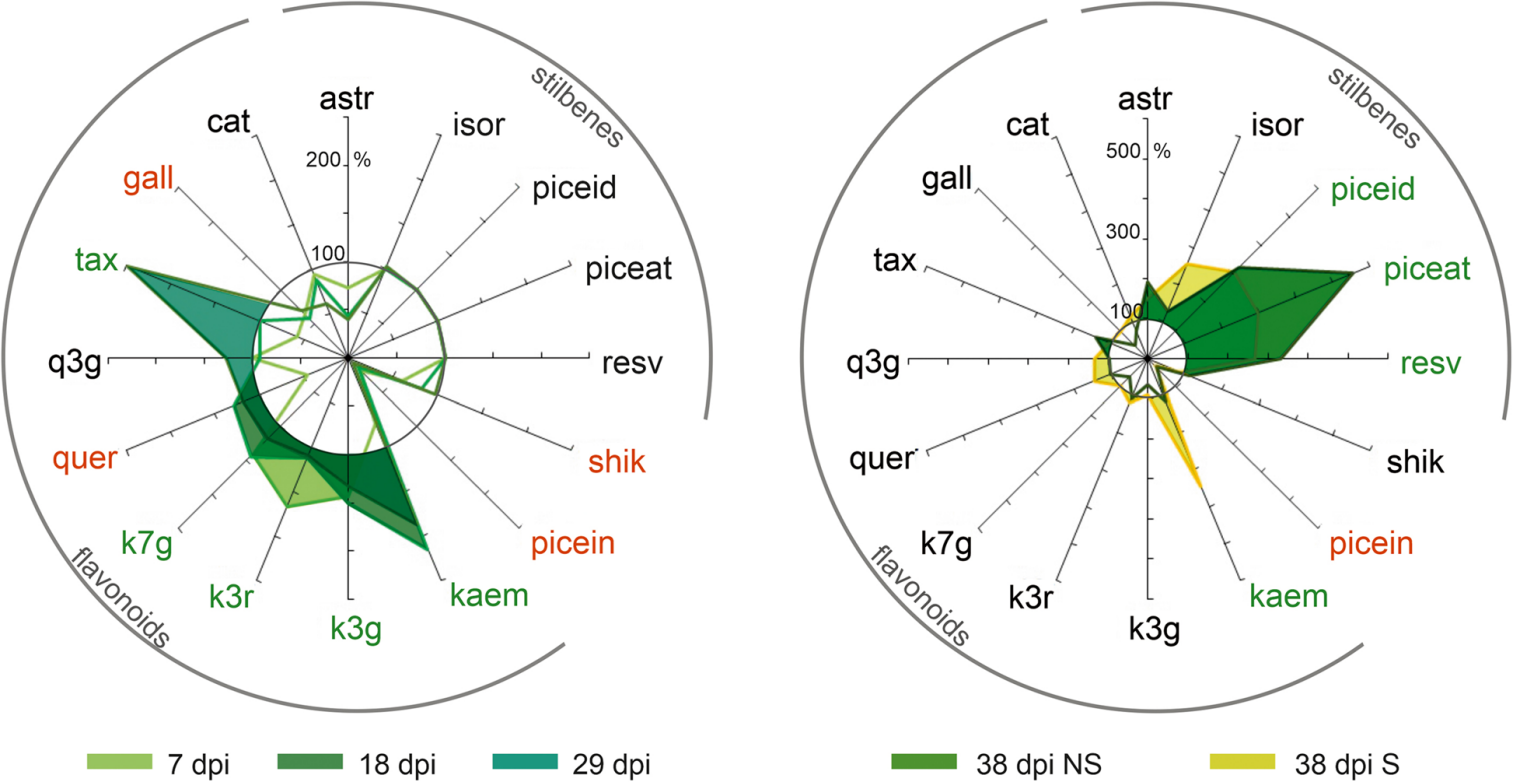
Phenolic compound contents in the resistant genotype PRA-R compared to the susceptible genotypes. Contents in PRA-R were expressed as percentage proportions (%) relative to the mean values for all three susceptible genotypes for all analysed time points. For 38 dpi, symptomatic (S) and non-symptomatic needles (NS) are shown. Increases are highlighted with shaded areas and significant changes compared to susceptible trees are indicated with green (increases) and red (decreases) compound names. Shown are astringin (astr), isorhapontin (isor), piceid, piceatannol (piceat), resveratrol (resv), shikimic acid (shik), picein, kaempferol (kaem), kaempferol 3-glucoside (k3g), kaempferol 3-rutinoside (k3r), kaempferol 7-glucoside (k7g), quercetin (quer), quercetin 3-glucoside (q3g), taxifolin (tax), gallocatechin (gall), and catechin (cat). Please note the different scaling at 38 dpi

Between 7 and 38 dpi and in all genotypes, the needle content for individual stilbenes, picein and taxifolin increased, remained constant for shikimic acid and catechin, and diminished for all remaining flavonoids (Fig. 10; Additional file 24: Figure S11), corresponding to known accumulation patterns during rust infection [12, 13]. At 38 dpi, the comparison of S and NS needles for each genotype revealed no significant differences in the needle phenolic profiles, except for the resistant genotype PRA-R, where higher contents of total flavonoids and of the individual compounds gallocatechin and catechin were found in S needles (Additional file 24: Figure S11; Additional file 25: Table S13).

The comparison of PRA-R with the three susceptible genotypes revealed significantly higher contents of the flavonoids kaempferol, its glucosides, and taxifolin in the resistant tree, particularly at early time points (Fig. 10). At 38 dpi, the contents of several stilbenes (piceid, piceatannol, resveratrol) were also higher (about 3- to fivefold) in both S and NS needles of the resistant tree. In contrast, needles of PRA-R had lower quercetin, gallocatechin and shikimic acid contents during early infection stages, and of picein during the entire period compared to the susceptible genotypes (Fig. 10; Additional file 24: Figure S11).

#### Plant hormones

**Fig. 11:**
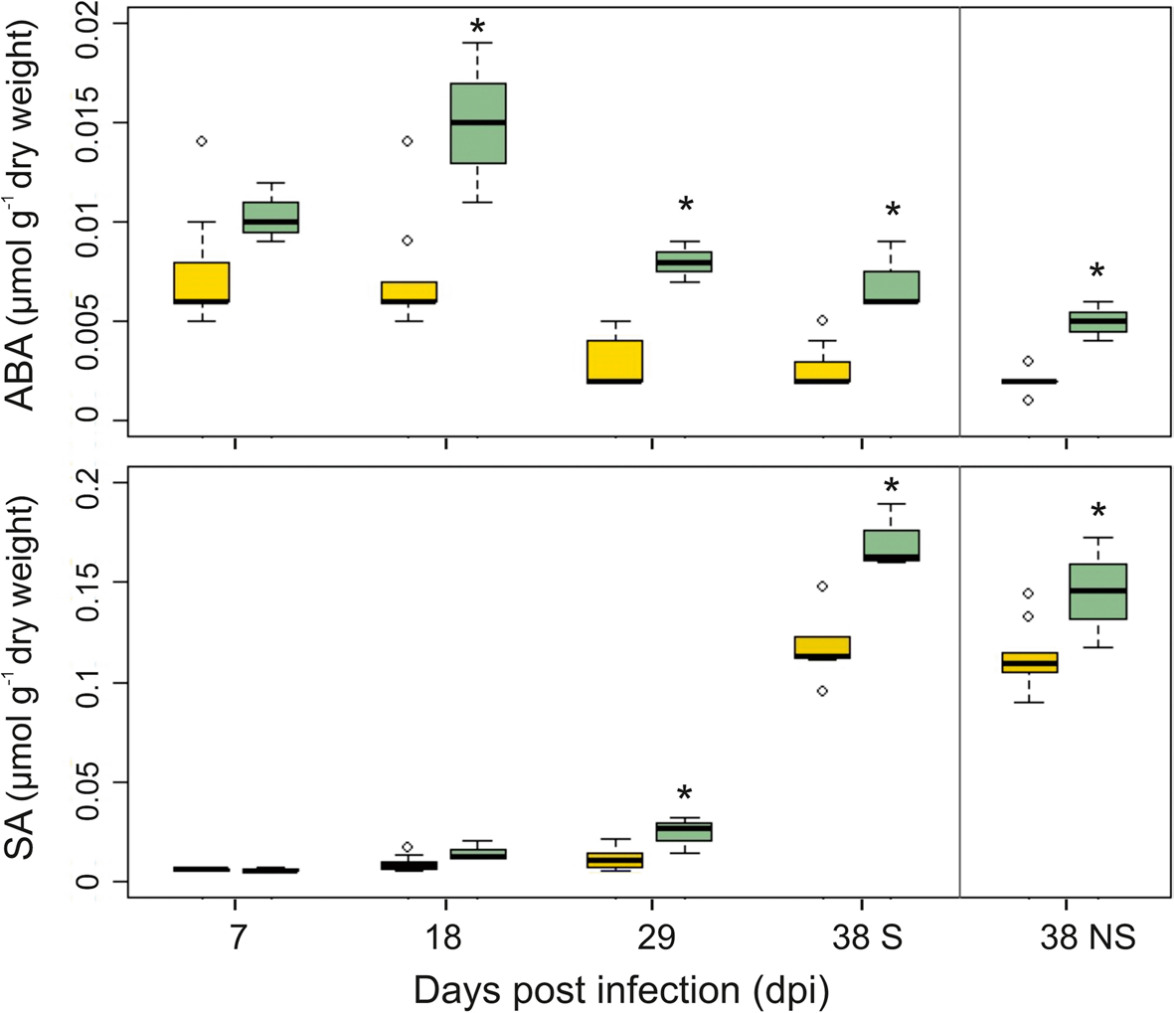
Abscisic acid (ABA) and salicylic acid (SA) content between 7 and 38 dpi in needles of susceptible genotypes (pooled data of PRA-A/B/D, yellow boxes) and of the resistant genotype PRA-R (green boxes). For 38 dpi, results for symptomatic (S) and non-symptomatic needles (NS) are shown. Three branches per tree were analysed; significantly different contents in PRA-R are marked with asterisks

Needle abscisic acid (ABA) and salicylic acid (SA) contents were higher in PRA-R than in the susceptible trees (significant for ABA from 18 dpi and for SA from 29 dpi onwards; Fig. 11), also in both S and NS needles at 38 dpi. In susceptible trees, ABA decreased and SA increased from 7 to 38 dpi, while in the resistant genotype ABA showed a distinct increase between 7 and 18 dpi (followed by a decrease). S and NS needles at 38 dpi did not differ in any of the analysed genotypes regarding their ABA and SA content.

## Discussion

In the present study, infection of Norway spruce by *C. rhododendri* was studied in trees of four genotypes growing in a subalpine forest with either susceptibility (PRA-A, PRA-B, PRA-D) or known enhanced resistance (PRA-R) to needle bladder rust. In agreement with previously conducted greenhouse experiments [12], a hypersensitive response (HR) to infection with *C. rhododendri* was observed in the resistant tree PRA-R, evidenced by tiny yellow–brown spots on the (overall rare) S needles. In contrast, substantial damage was recorded for the susceptible genotypes, with a higher percentage of S needles and severe symptoms (as shown in Fig. 2).

Symptoms of infection were clearly visible at 38 dpi, enabling a separate sampling of S and NS needles. 38 dpi NS needles were used as control sample for S needles within each genotype and to compare constitutive differences between the genotypes. Both is based on the fact that NS needles of infected trees mainly correspond to control needles of unaffected trees (i.e. show no infection-induced changes; [12]) and thus can serve as control samples in nature, where no unaffected trees of the same genotype are available.

We thus explored the main differences found for induced defence (38 dpi S needles vs NS needles within each genotype) and constitutive defence (38 dpi PRA-R NS needles vs PRA-A/B/D NS needles) for the most relevant defence pathways found by [12]. Moreover, RT-qPCR (performed for a set of genes involved in both, induced and constitutive defence) confirmed most of the DEGs found by transcriptomic analysis, and allowed us to explore differences between the resistant and susceptible trees at earlier time points (7, 18 and 29 dpi needles) which result from a combined effect of induced and constitutive defence as needles at this infection stages could not be split into S and NS needles.

At 38 dpi, RNA-Seq of S needles was consistent with the known induction of defence responses previously described for young clones of the genotype ASS-7 [12]. This includes equal proportion of over- and under-expressed DEGs, and significantly enriched GO terms and enzymes mapped in KEGG pathways. In addition, pathway fingerprints showed that process dynamics *in natura* (38 dpi) were very similar to developments in the greenhouse experiment (39 dpi), but some specific DEGs coding for enzymes previously proposed to be involved in plant defence pathways differed in their performance in comparison to ASS-7 [12]. Importantly, the small proportion of symptomatic tissue in PRA-R S needles resulted in a comparably small set of DEGs in the resistant tree.

Differences in gene expression between PRA-R and susceptible genotypes were found at early infection stages as well as in 38 dpi S and NS needles, including several genes clearly related to defence mechanisms against fungi. The relevance of constitutive differences across genotypes for spruce resistance to needle rust is shown by the high number of shared DEGs (706) found by comparing 38 dpi NS needles of PRA-R with those of the three susceptible genotypes (Fig. 5). Even though the limited number of analysed trees and genetic diversity across studied trees could affect the identification of constitutive differences related to pathogen resistance, the use of closely located trees is expected to diminish this bias due to their assumed relatedness (see also [18]). In addition, several of the identified over-expressed DEGs were previously identified to be involved in pathogen defense and plant rust resistance (Additional file 12: Table S7), underlining the significance of the analysis performed. Indeed, enriched GO terms for over-expressed genes, such as response to stress, biotic stimulus and kinase activity, suggest that PRA-R had a higher level of constitutively expressed defence-related enzymes and enhanced pre-formed barriers that partially or completely protect the needle from pathogen invasion and spreading. Additional shared differences between PRA-R and susceptible genotypes in the induced defence and at early infection stages (Fig. 5, 6, 7; Additional file 18: Figure S8) indicate that the enhanced resistance of the genotype PRA-R is probably based on a combination of inducible and constitutive defence mechanisms. This strategy was already shown to determine the efficiency of the plant defence to biotic attack [39] as well as differences between genotypes, as previously reported, for example, for pine trees [26, 40]. However, our results contradict the hypothesis that long-living organisms rely mainly on systemic-induced resistance to respond to pathogens [25].

### Plant-pathogen interaction

#### 38 dpi induced defence

At 38 dpi, when pronounced infection symptoms were observed, genes coding for several enzymes implicated in plant-pathogen interaction were differentially regulated in S needles of all genotypes (Additional file 7: Table S4; Additional file 9: Table S6) in a similar way as previously described for ASS-7 [12]. For Norway spruce, an induced defence through pathogen-associated molecular pattern (PAMP)-triggered immunity (PTI) and effector-triggered immunity (ETI) against fungal pathogens including stomatal closure, HR and cell wall reinforcement has been reported [12, 41, 42]. Accordingly, exploring PTI and ETI at 38 dpi revealed induced over-expression of the calmodulin gene MA_52889g0010, the calcium-binding protein gene MA_10431324g0020, and the enhanced disease susceptibility 1 gene MA_101621g0020 in all genotypes (Additional file 18: Figure S8). However, in the susceptible genotypes, down-regulation of other calmodulins (e.g., CALM; MA_10204459g0010), calcium-binding proteins (e.g., CML; MA_10207337g0010) and a respiratory burst oxidase (RBOH; MA_10427209g0020) could have disrupted the Ca^2+^ signalling pathway and thereby the defence response, enabling *C. rhododendri* to continue colonizing needle tissues (see also [43–45]).

In PRA-B and PRA-D, down-regulation of a WRKY transcription factor 2, MA_16118g0010, could contribute to a reduced stimulation of defence-related genes and programmed cell death in these genotypes compared to PRA-R. Most WRKY proteins are involved in the response of plants to bacterial and fungal pathogens; they usually mediate signalling by the elicitor molecule encoded by the pathogen and quickly induce programmed cell death of infected cells to avoid posterior invasion [46, 47].

#### 38 dpi constitutive defence

Instead, constitutive higher expression of the calmodulin gene MA_52889g0010 in PRA-R could have enhanced the activation of the defence response in this genotype, also by promoting defence-related gene induction and supporting the expression of two pathogenesis-related proteins 1 (PR1), MA_501572g0010, MA_53673g0010 [12]. However, RT-qPCR did not confirm the induction of MA_53673g0010 for all genotypes, but lower expression levels for PRA-R from 29 dpi onwards (Additional file 18: Figure S8). Further experimental validation is required before an antifungal function can be attributed to these PR-1 proteins [48].

Moreover, four ketoacyl-CoA synthases (KCS) genes, MA_105806g0010, MA_10605g0020, MA_46113g0010, MA_759169g0010, had constitutively reduced expression in PRA-R compared to susceptible genotypes. These genes are involved in very-long-chain fatty acid (VLCFA) biosynthesis. These VLCFAs containing 22 or more carbons are required for cuticula formation and are considered important signalling molecules for fungi to recognize their hosts [49] and to initiate pre-penetration processes, such as conidia germination and appressoria development. For example, silencing of the wheat 3-ketoacyl-CoA synthase gene (TaKCS6; Norway spruce ortholog MA_10605g0020) led to a reduction in cuticular wax load and attenuated conidia germination of *Blumeria graminis* f. sp. *tritici* [50]. Future research characterizing the function of those genes and specific wax components is needed to understand their roles in the regulation of plant-pathogen interaction in general [51, 52] and in the specific case of spruce enhanced resistance to needle rust.

### MAPK signalling pathway and plant hormone signal transduction

#### 38 dpi induced defence

Differential expression patterns observed for MAPK and hormone signalling pathways were very similar to those reported for infected young clones under greenhouse conditions [12]. In addition, significantly higher contents of ABA and SA, both of which can be involved in triggering induced defence and stress adaptation [53–56], were observed in PRA-R compared to the susceptible genotypes along the entire time course (Fig. 11). The expression of several key defence genes of Norway spruce is greatly influenced by these plant hormones, including mitogen-activated protein kinase 6 (MPK6), MA_10437020g0010 [57, 58] or transcription factor MYC2, MA_10435905g0020 [59, 60]. The over-expression of these genes in combination with some others influenced by ethylene (e.g., MKK9: MA_10194g0020; [61]) promotes defence responses *via* expression of several antimicrobial peptides that are efficient against fungi, such as basic endochitinases B (MA_10313114g0010; MA_8921185g0010), plant defensin (MA_1489g0010; [62]) and class IV chitinase (MA_10427514g0010).

#### 38 dpi constitutive defence

These induced defence responses probably act in concert with higher constitutive levels of the basic endochitinase B (MA_10313114g0010), antifungal peptides (salt stress/antifungal: MA_10430127G0010; chitinase: MA_7544918g0010) or signalling proteins (toll-interleukin-resistance (TIR) protein: MA_3160g0010; leaf rust 20 disease-resistance locus receptor-like protein kinase: MA_6204656g0010) in PRA-R needles. Even at early stages of infection, they could confer enhanced resistance against chitin-containing fungal pathogens.

A reduced constitutive expression of the JASMONATE-ZIM-DOMAIN (JAZ) repressor gene (MA_10427283g0030) was observed at 38 dpi in PRA-R, which can accelerate its proteasomal degradation in comparison with the susceptible genotypes, thereby relieving repression of MYC2 (MA_10435905g0020) activity and thus allowing the expression of target defence genes controlled by this master regulator [60, 63]. ABA signalling can also influence disease resistance by regulating stomatal closure, impeding pathogen entry into the plant [64]. Thus, low ABA levels found in the susceptible genotypes could contribute to the infection success, but it remains unclear whether low content levels are intrinsic/genetically programmed or caused by the pathogen [65]. Finally, the higher level of SA in PRA-R needles may be another reason for the limited damage seen in S needles in comparison with susceptible genotypes (see [66, 67]), as SA plays a critical role in regulating HR and cell death [56].

### Primary and secondary metabolism

Both, primary and secondary metabolism, was strongly affected in S needles of all genotypes at 38 dpi (Additional file 10: Figure S4). The biosynthesis of protective metabolites can come at the cost of a down-regulation of enzymes involved in photosynthesis, as previously described for ASS-7 [12] and in accordance with the reduced photosynthetic activity and chlorophyll contents in infected needles [9, 10]. By contrast, starch and sucrose metabolism pathways were stimulated during the infection process, indicative of a shift from photosynthetic carbon assimilation to the consumption of stored resources. Many induced and constitutive DEGs were involved in the biosynthesis of secondary metabolites such as terpenes and phenolic compounds, whose antifungal properties are pivotal to conifer response to fungal pathogens [12, 13, 15, 68]. Indeed, the metabolite profiles of the resistant and susceptible genotypes clearly differed, most likely contributing to their different performance in response to needle bladder rust.

### Terpenes

The roles of terpenes in conifer pathogen defence are well documented [14] and in the current study, clear differences between genotypes were found in early stages of infection for the analysed comprehensive set of monoterpenes and sesquiterpenes (low molecular weight terpenes that are volatile at ambient conditions). Overall, the resistant genotype PRA-R had lower terpene contents than the susceptible genotypes: monoterpenes were decreased at earlier times (7 dpi), whereas sesquiterpenes remained lower during the entire observation period (Fig. 8). These findings were supported by higher expression levels of several sesquiterpene synthases (MA_10434494g0010; MA_10435334g0020) in all susceptible genotypes in comparison to PRA-R since the very beginning of the infection process, and by the induced over-expression of a farnesyl diphosphate synthase (MA_175884g001), which was observed only in susceptible genotypes at 38 dpi (Additional file 18: Figure S8). In agreement with data reported for greenhouse conditions [12], the mevalonate pathway (occurring in the cytoplasm) was enhanced in comparison to the non-mevalonate pathway (occurring in the plastids) in S needles at 38 dpi. Of note, two metabolites were consistently less abundant in PRA-R than in the susceptible genotypes, namely 3-carene and β-farnesene (Additional file 23: Figure S10). 3-carene has received considerable interest as a defence-related compound in spruce, as it has been related to pine resistance to the white pine weevil [69] and was shown to be induced by methyl jasmonate [70, 71]. A possible explanation for the generally lower terpene production in the resistant genotype could be related to the associated metabolic cost [72] and a shift to other metabolic pathways that are more effective against needle bladder rust. It appears that there are trade-offs between the mevalonate or GAP-pyruvate pathways, which produce terpenes, and the shikimic acid pathway, which produces phenolic compounds [14, 73, 74]. This could also explain the decreased contents of mono- and sesquiterpenes in S needles of all genotypes in favour of phenolic compound production. However, additional analyses including a higher number of trees are needed to exclude random genotype-related differences (see also [22]). In contrast, a few terpenes that remained consistently higher in the resistant genotype over time were previously identified as effective antifungal compounds, such as geranyl acetate [75]. The increase of the apocarotenoids geranyl acetone and α-ionone (Additional file 23: Figure S10) in PRA-R could have resulted from differential regulation of the carotenoid metabolism. In our study, a Carotenoid Cleavage Dioxygenase 1 (CCD1; MA_10435932g0010) had a higher constitutive expression in PRA-R compared with at least two susceptible genotypes (PRA-B and PRA-D), consistent with higher contents of these compounds. Several apocarotenoids have antifungal and antimicrobial properties, but also important signalling functions (reviewed by [76]).

### Phenolics

Variations in the phenolic needle profiles among genotypes were detected from the very beginning of the infection. The content of shikimic acid, a precursor of all studied compounds (flavonoids, stilbenes, picein), was lower in the resistant genotype (PRA-R) from 7 to 18 dpi, possibly due to its use in the production of specific flavonoids (kaempferol, its glucosides, and taxifolin). When symptoms were pronounced at 38 dpi, additional priority was apparently given in the resistant tree to specific stilbenes (piceid, piceatannol, resveratrol), both in S and NS needles. Importantly, a set of DEGs related to phenylpropanoid biosynthesis was induced in S needles (38 dpi) for all genotypes in agreement with previous observations [12] and studies on fungal wood and root infections (see, e.g., [53, 77]). In addition, significant constitutive (in NS needles at 38 dpi) gene expression differences between PRA-R and the susceptible genotypes were observed (Additional file 15: Figure S7 B). For instance, the constitutively lower expression of peroxidases (MA_20223g0020, MA_8643639g0010) and cinnamoyl-CoA reductase (CCR; MA_166604g0010) together with a higher expression of a 4-coumarate-CoA ligase (4CL; MA_8938767g0010) in PRA-R can feed into flavonoid and stilbenoid biosynthetic pathways with more 4-coumaroyl-CoA in comparison to the susceptible genotypes. In addition, the lower expression of the peroxidase MA_66201g001 and the cinnamyl-alcohol dehydrogenase MA_10432110g0010 in PRA-R at early stages of infection could imply that most of available resources are directed to the production of flavonoids and stilbenes instead of simple phenylpropanoids like picein (Additional file 18: Figure S8).

Also, several genes related to the flavonoid and stilbenoid biosynthetic pathways differed constitutively between the resistant and susceptible genotypes at 38 dpi (Additional file 15: Figure S7 B). Five enzymes previously associated with Norway spruce defence mechanisms [78] were over-expressed in PRA-R, namely caffeoyl-CoA O-methyltransferase (CCOAMT; MA_9065834g0010), chalcone synthase (CHS: MA_10434819g0010), shikimate O-hydroxycinnamoyltransferase (HCT; MA_106573g0010), bifunctional dihydroflavonol 4-reductase/flavanone 4-reductase (DFR; MA_79460g0010), and flavonoid 3',5'-hydroxylase (F3´5´H/CYP75A: MA_99372g0010). In addition, the under-expression of the anthocyanidin reductase (ANR; MA_203441g0010) in PRA-R could prevent direct resources towards flavan-3-ols biosynthesis instead of flavonols and stilbenes, resulting in higher concentrations in the resistant genotype needles. RT-qPCR proved that differences among genotypes were maintained from 7 dpi onwards (e.g., F3´5´H/CYP75A; MA_99372g0010), probably leading to the specific phenolic profile of PRA-R that could have contributed to the higher taxifolin content in PRA-R needles, a flavonoid with strong antifungal properties that can be induced by various pathogens [13, 35].

With regard to the induced response, over-expression of a flavonoid 3`-monooxygenase (F3´H/CYP75B1: MA_76780g0010) and under-expression of a flavonol synthase (FLS: MA_4711g001) in S needles of all susceptible genotypes was consistent with a higher kaempferol content in S needles of the resistant tree PRA-R (Fig. 10). In addition, the chalcone synthase (MA_5735g0010) previously reported by [12] was not induced in most of the susceptible genotypes, and showed lower expression in PRA-R across all time points which can contribute to its higher stilbenes content. Finally, flavanone-3-hydroxylase (F3H) and flavonoid 3´,5´-hydroxylase (F3´5´H; MA_10420573g0010), which play important roles in the biosynthesis of phenolic compounds involved in spruce defence against the bark beetle-associated fungus *Endoconidiophora polonica* [35, 36] were not consistently differentially expressed in the current study.

## Conclusion

By a combination of transcriptional and secondary metabolite analyses, the study showed that (1) the defence response to needle bladder rust in adult spruce trees growing in a forest corresponds well to results previously reported for experimentally infected young clone trees grown in a greenhouse, both regarding the timing and development of infection and genes and metabolic pathways involved. Moreover, the comparison of different genotypes growing next to each other including one resistant tree (PRA-R) underpinned that (2) several previously identified key genes of spruce defence to fungal attack are differently regulated in PRA-R, both constitutively (in NS needles) and infection-induced (in S needles). Importantly, (3) genetic and metabolic differences between the resistant and susceptible genotypes appeared at all analysed infection stages (7 to 38 dpi) and probably (4) result in a more effective HR in PRA-R, which can reduce penetration and spread of the needle rust fungus *via* localized plant cell death at the site of infection. Identified genes and related proteins, hormones and secondary metabolites should be further explored as potential markers for spruce resistance to *C.* *rhododendri*, which may enable the screening of large populations for resistant genotypes and support current reproduction programs for resistant plant material. In addition, the verification and location of related proteins (i.e., by western blots and immunohistochemistry) could further refine our knowledge of the complex resistance mechanisms in Norway spruce against foliar pathogens.

## Methods

### Plant material and experimental design

Adult Norway spruce trees from a subalpine spruce forest near Innsbruck (Praxmar, 1614 m s. l., N 47°09.495, E 11°08.201) were used. The site has been highly affected by needle rust infections for decades. The tree PRA-R is one of the extremely rare trees with hardly any susceptibility to needle rust, hereafter termed “resistant” and was already used in previous studies [9, 11, 13]. Three surrounding trees of similar size and age with high infection intensities over the last years (PRA-A, PRA-B and PRA-D; Additional file 1: Table S1) were chosen as control trees. Close location of all trees was important to ensure equal environmental and soil conditions as well as exposition to rust spores.

The research on Norway spruce complies with the IUCN Policy Statement on Research Involving Species at Risk of Extinction and the Convention on the Trade in Endangered Species of Wild Fauna and Flora. Norway spruce is a common and widespread forest tree and is not subject of specific legislation. The study and research protocol comply with all relevant institutional, national and international guidelines and legislation. The local agricultural community ('Agrargemeinschaft Praxmar') permitted collection of branches from the selected trees in the study area. Study trees were located within a managed forest composed of Norway spruce and *Pinus cembra* only; formal identification of the trees was conducted by A. Ganthaler (deposition of a voucher specimen not applicable).

Needles were sampled 7, 18, 29, and 38 days after bud break, hereafter defined as days post infection (dpi). Bud break and complete shedding of the bud scale and the limpid membrane covering the bud at early stages represent the start of infection, as spores, present in this period in the air, can enter the needles [7]. Sampling times were selected based on our previous study [12]. One current-year shoot was collected from each of three selected and marked branches on each tree, at each sampling (corresponds to three replicates per tree and time point; from different parts of the crown). The needles from each shoot were cut and divided into four cryo vials (for RNA-Seq/RT-qPCR, phenolics/hormones, terpenes, and backup). Vials were immediately placed in a liquid nitrogen dry shipper and stored at -80 °C in the laboratory until further analysis. At 38 dpi, samples were further subdivided into symptomatic (S) and non-symptomatic (NS) needles prior to freezing (Fig. 2), as infection symptoms were clearly visible in all genotypes to the naked eye. While S needles of susceptible trees were highly affected showing discoloration and several spore stocks, S needles of the resistant tree had only some yellow spots.

We firstly investigated *via* RNA-Seq the induced defence response for each genotype by exploring the DEGs in S compared to NS needles at 38 dpi (for detailed study design see Fig. 1). The use of NS needles from the respective tree as reference enabled us to focus on the changes in expression patterns in S needles. Secondly, constitutive defence mechanisms were investigated by comparing gene expression in NS needles of the resistant genotype (PRA-R) with that in NS needles of the susceptible genotypes. In addition, at all stages of infection, defence mechanisms were explored by RT-qPCR for a set of 30 selected genes. Finally, differences in gene expression within and among genotypes were related to changes in specific metabolite levels.

### RNA isolation, sequencing, and mapping to the reference genome

RNA was extracted by homogenizing needles with mortar and pestle and subsequently mixing with lysis solution in order to preserve RNA integrity. The Protocol B Sigma-Aldrich Spectrum Plant Total RNA Kit (PN: STRN50-1KT) was used for extraction according to the manufacturer’s guidelines (Sigma Aldrich, St. Louis, USA). RNA concentrations were measured with a NanoDrop 2000c spectrophotometer (Thermo Fisher Scientific, Waltham MA, USA) and approximately 1 µg of RNA was treated with DNAse I in a reaction volume of 10 µl. Quality of DNAse I-treated total RNA was determined by recording electropherograms on a Fragment Analyzer (RNA-Seq samples; 38 dpi) or a 4200 TapeStation system (RT-qPCR samples; 7 to 29 dpi; both Agilent technologies, Santa Clara CA, USA). On samples for RNA-Seq, 5 µl of DNAse I digested total RNA was processed with QuantSeq 3' mRNA-Seq FWD Library Preparation Kit and each reaction was spiked in with SIRV-Set 3 variant controls by following the manufacturer’s guidelines (Lexogen, Vienna, Austria). Quality of the libraries was determined using a Fragment Analyzer (Agilent technologies, Santa Clara, USA). Samples were pooled in equimolar ratio and the library pool was quantified using a Qubit dsDNA HS assay kit (Thermo Fisher Scientific, Waltham MA, USA). Sequencing was performed on an Illumina NextSeq 500 system with SR75 High Output Kit at Lexogen (Lexogen, Vienna, Austria). The obtained reads were mapped to the Norway spruce reference genome [79] using the open-source software STAR [80]. We used the alternate protocol 1 as described in [80] and only those contigs that had a gene annotation in the Norway spruce genome assembly were retained for mapping. Since the Norway spruce reference genome is approximately 20 GB in size, genome indices were produced by using a 189 GB RAM server according to the required Genome size/bytes ratio described in [80]. The detailed STAR settings used for mapping and genome index generation can be found in the Additional file 26: Command S1.

### Differential gene expression analysis at 38 dpi

For differential gene expression analysis, the DESeq2 package [81], which is implemented in the *Bioconductor* platform in R [82], was used. Briefly, DESeq2 transforms read counts per gene obtained from RNA-Seq data into systematic contrasts of gene expression across experimental conditions. For this, DESeq2 uses so called shrinkage estimators which were shown to better estimate and compare fold changes in gene expression across treatments compared to other available methods [81]. The null hypothesis tested implies that the logarithmic fold change in gene expression between contrasted samples is exactly zero. We first used the *DESeqDataSetFromHTSeqCount* function for importing read count data and pre-filtered for rows with zero counts. Subsequently, differential gene expression analysis was performed by using the *DESeq* function, which generates the log2fold changes with adjusted *p*-values for each contrast. DESeq2 applies a FDR cutoff, in this case 0.1, based on the reported p-adjusted values (padj), to detect DEG.

Venn diagrams of over- and under-expressed transcripts were created by using jvenn [83]. Information from the ConGenIE database (http://congenie.org/enrichment; [84]) provides useful information of the ongoing Norway spruce sequencing project, even though genes are not annotated yet hindering the work of data mining. Nevertheless, gene role or function can be investigated further by using as starting point its PFAM domains or GO terms. GO enrichment analysis with Fisher exact tests on differentially expressed transcripts, metabolic pathway and orthology-oriented functional annotations of the protein sequences were conducted following the protocols previously described in [12] (Additional file 27: Table S14; Additional file 28: Table S15). These analyses were applied for differentially over- and under-expressed genes (DEGs). Orange Data Mining (https://orange.biolab.si; [85]) was used to plot heat maps as a graphical method for visualizing up-/down-regulated pathways and genes among samples. Columns (samples) and rows (pathways/genes) were sorted by clustering (clusters data by similarity). Heat map uses k-means for grouping and hierarchical clustering with average linkage for clustering (ordering) rows. Here, Euclidean distance was used as distance metric.

### RT-qPCR

RT-qPCR was conducted as previously described in [12]. Primer sequences are listed in Additional file 29: Table S16 [12, 35, 36, 86, 87]. Two reference genes (RGs), actin and ubiquitin were included for normalization. Both genes were previously found to be suitable RGs for *C. rhododendri* infection studies in Norway spruce [12], but ubiquitin was excluded from the analysis due to its unequal expression among the investigated genotypes (Additional file 30: Figure S12). Amplification efficiency-corrected target gene Cq values were normalized to actin and expression changes were calculated using the 2^−ΔΔCT^ method [88]. Mean Cq and RNA integrity numbers are enclosed in Additional file 19: Table S11. PCA plots by using mean ΔCq values were generated with ClustVis [89].

Statistical analyses (two-tailed unpaired t-test with Welch’s correction) of the normalized expression data (∆Cq values) were calculated with GraphPad Prism 5 software (GraphPad Software, San Diego, CA, USA). A *p*-value < 0.05 was considered as significant.

### Identification and quantification of volatile terpenes

Terpene analysis was performed at the Institute for Plant Molecular and Cell Biology (IBMCP-CSIC-UPV, Valencia, Spain) Metabolomics Platform. Spruce needles were frozen in liquid nitrogen and ground to fine powder. 100 mg samples of frozen powder were resuspended in 1 mL of a saturated NaCl solution, sonicated for 5 min and subjected to headspace solid phase microextraction (HS-SPME), using a 65 µM PDMS/DVB (Supelco, Bellefonte, PA, USA) fibre. Equal amounts of all samples were pooled to create a reference mix, which was injected every 10 samples. Pre-incubation and extraction were performed at 50 ºC for 10 and 20 min, respectively. Desorption was performed for 1 min at 250 ºC in splitless mode. Volatile terpenes trapped on the fibre were analysed by GC–MS using a COMBI PAL autosampler (CTC Analytics, Zwingen, Switzerland), a 6890 N gas chromatograph (Agilent Technologies, Santa Clara, CA, USA) and a 5975B Inert XL MSD mass spectrometer (Agilent). The analytes were separated on an Agilent J&W Scientific DB-5 fused silica capillary column (5%-phenyl-95%-dimethylpolysiloxane as stationary phase, 60 m length, 0.25 mm i.d., and 1 μm film thickness). Oven temperature conditions were 40 ºC for 2 min, 5 ºC min^−1^ ramp up to 250 ºC, which was then held isothermally at 250 ºC for 5 min. Helium was used as carrier gas at 1.4 ml min^−1^ constant flow. Mass/z detection was achieved using an Agilent mass spectrometer operated in the EI mode (ionization energy 70 eV; source temperature 230 ºC). Data acquisition was performed in the scanning mode (mass range m/z 35–220). Chromatograms and spectra were recorded and processed using the Enhanced ChemStation software for GC–MS (Agilent). Compound identification was based on both the comparison between the mass spectrum of each putative compound with those of the NIST 2005 Mass Spectral library and the match to a GC retention time and Mass Spectra custom library generated using commercially available compounds. The principal component analysis and hierarchical clustering were performed with MetaboAnalyst 5.0 (https://www.metaboanalyst.ca/). After generalized logarithm transformation, data scaling was performed by mean-centering and dividing by the square root of the standard deviation of each variable. Hierarchical clustering was done using Ward clustering algorithm and Euclidean distance measure.

### Identification and quantification of phenolic compounds and hormones

Sample preparation, extraction and analysis of phenolic metabolites were conducted as described in [13], adapted to include hormone analysis modified after [90]. Briefly, needles were freeze-dried, homogenized, and extracted two times with 1 ml of 95% (v/v) ethanol, containing orientin, naringin, and ABA-d6 as internal quantification standards. Eleven flavonoids (kaempferol, kaempferol 3-glucoside, kaempferol 7-glucoside, kaempferol 3-rutinoside, quercetin, quercetin 3-glucoside, quercitrin, naringenin, taxifolin, catechin, gallocatechin), five stilbenes (astringin, isorhapontin, piceid, piceatannol, resveratrol), three simple phenylpropanoids (picein, gallic acid, chlorogenic acid), one precursor (shikimic acid) and three plant hormones, namely abscisic acid (ABA), salicylic acid (SA) and jasmonic acid (JA), were identified and quantified by liquid chromatography-mass spectrometry (UHPLC-MS). We used an Eksigent ultraLC 100 UHPLC system with a reversed-phase column (NUCLEODUR C18 Pyramid, EC 50/2, 50 × 2 mm, 1.8 μm, Macherey–Nagel, Düren, Germany), coupled to a QTRAP 4500 mass spectrometer (AB SCIEX, Framingham, MA, USA) operated in negative ion mode using multiple reaction monitoring (MRM). Based on retention time and MRM transition (for detailed parameters see [13] and [90]), and calibration curves of authentic samples of all substances, peaks were automatically detected and normalized relative to internal standards, and concentrations were calculated using the software Analyst (version 1.7) and SCIEX OS-MQ (version 1.6.1, both AB SCIEX, Framingham, MA, USA).

The compounds naringenin, quercitrin, gallic acid, chlorogenic acid, and JA exhibited concentrations below the individual limit of quantification (between 0.001 and 0.027 µmol g^−1^) at all selected time points and thus, were not included in result tables and further analysis. Differences were tested (1) between the resistant tree and susceptible trees for all time points, and (2) between S and NS needles at 38 dpi with analyses of variance (ANOVA), after first assuring that data satisfied the Kolmogorov–Smirnov test for normality; pairwise tests were then conducted using Bonferroni or Tamhane tests, depending on their satisfying or not, respectively, the Levene’s test for equal variance. All tests (two-tailed) were performed pairwise at a probability level of 5% using SPSS (version 24; SPSS, IL, USA), and all values are given as mean ± standard error (SE).

## Supplementary Information

Below is the link to the electronic supplementary material.

Additional file 1: **Table S1.** Studied trees.

Additional file 2: **Table S2.** Summary of RNA-sequencing and mapping results.

Additional file 3: **Figure S1.** Inter-replicate correlation plots.

Additional file 4: **Table S3.** Differentially expressed genes.

Additional file 5: **Figure S2.** Numbers and proportion of differentially expressed genes.

Additional file 6: **Figure S3.** Induced defence: Gene ontology (GO) term enrichment analysis

Additional file 7: **Table S4.** Induced defence: KEGG Orthology (KO) assignment and proportion.

Additional file 8: **Table S5.** Induced defence: KEGG pathways of differentially expressed genes by *P. abies* infected by *C. rhododendri* at 38 dpi.

Additional file 9: **Table S6.** Induced defence: KEGG Orthology (KO) detailed output.

Additional file 10: **Figure S4.** Induced defence: Heat map plot for DEGs distribution among pathways (pathway fingerprint).

Additional file 11: **Figure S5.** Induced defence: Heat map plot for DEGs belonging to defence pathways.

Additional file 12: **Table S7.** PRA-R constitutive defence: DEGs shared by all contrasts between PRA-R non-symptomatic needles (PRA-Rn) and PRA-A/B/D non-symptomatic needles (PRA-ABDn) at 38 dpi.

Additional file 13: **Figure S6.** Gene ontology (GO) term enrichment analysis for DEGs in PRA-R non-symptomatic needles (NS) contrasted to NS needles from all susceptible genotypes.

Additional file 14: **Table S8.** KEGG Orthology (KO) assignment and proportion for DEGs in PRA-R non-symptomatic (NS) needles contrasted to NS needles from all susceptible genotypes.

Additional file 15: **Figure S7.** Constitutive differences between the resistant and susceptible genotypes viewed through heat maps of DEGs distribution across pathways (pathway fingerprint).

Additional file 16: **Table S9.** KEGG pathways of differentially expressed genes in PRA-R non-symptomatic (NS) needles contrasted to NS needles from all susceptible genotypes.

Additional file 17: **Table S10.** KEGG Orthology (KO) detailed output for DEGs in PRA-R non-symptomatic (NS) needles contrasted to NS needles from all susceptible genotypes.

Additional file 18: **Figure S8.** RT-qPCR results of selected differentially expressed transcripts.

Additional file 19: **Table S11.** RNA integrity numbers and RT-qPCR data.

Additional file 20: **Animation S1.** Animation on the principal component analysis (PCA) of RT-qPCR analysed transcripts for all samples. Note the high conformance within genotypes and replicates (1–3) with a clear separation between resistant and susceptible genotypes and between symptomatic (S) and non- symptomatic (NS) needles. For static version see Fig. 7. At 38 dpi, S needles (As, Bs, Ds, Rs) are represented by an additional colour in contrast to the NS needles (A, B, D, R). Values from 38 dpi were virtually kept till the day 50^th^ to make them visible for a while on the animated version of the plot.

Additional file 21: **Table S12.** Relative levels of terpenes identified in P. abies needles across time and genotype.

Additional file 22: **Figure S9.** Principal component analysis (PCA) of terpene contents over time.

Additional file 23: **Figure S10.** Terpenes with different content levels in the resistant genotype PRA-R compared to susceptible genotypes at all time points.

Additional file 24: **Figure S11.** Content levels of all individual phenolic compounds.

Additional file 25: **Table S13.** Raw data LC–MS analysis.

Additional file 26: **Command S1.** STAR command.

Additional file 27: **Table S14.** Proportion of GO-terms associated DEGs.

Additional file 28: **Table S15.** Sequence datasets of plant species applied for KEGG Orthology (KO) assignment using the KEGG Automatic Annotation Server (KAAS)

Additional file 29: **Table S16.** RT-qPCR assay details.

Additional file 30: **Figure S12.** Two reference genes (RGs), actin and ubiquitin included for normalization.

## Data Availability

Raw sequence data have been submitted to the NCBI Short Read Archive (SRA) under accession number PRJNA783365.
