# Supplementary material for: Advances in understanding Norway spruce natural resistance to needle bladder rust infection: transcriptional and secondary metabolites profiling
Source: BMC Genomics. 2022 Jun 13;23:435. doi: 10.1186/s12864-022-08661-y (PMC9190139; doi:10.1186/s12864-022-08661-y)
Supplement: Supplementary file 3 — Additional file 3: Figure S1. Inter-replicate correlation plots. [file 12864_2022_8661_MOESM3_ESM.pdf]

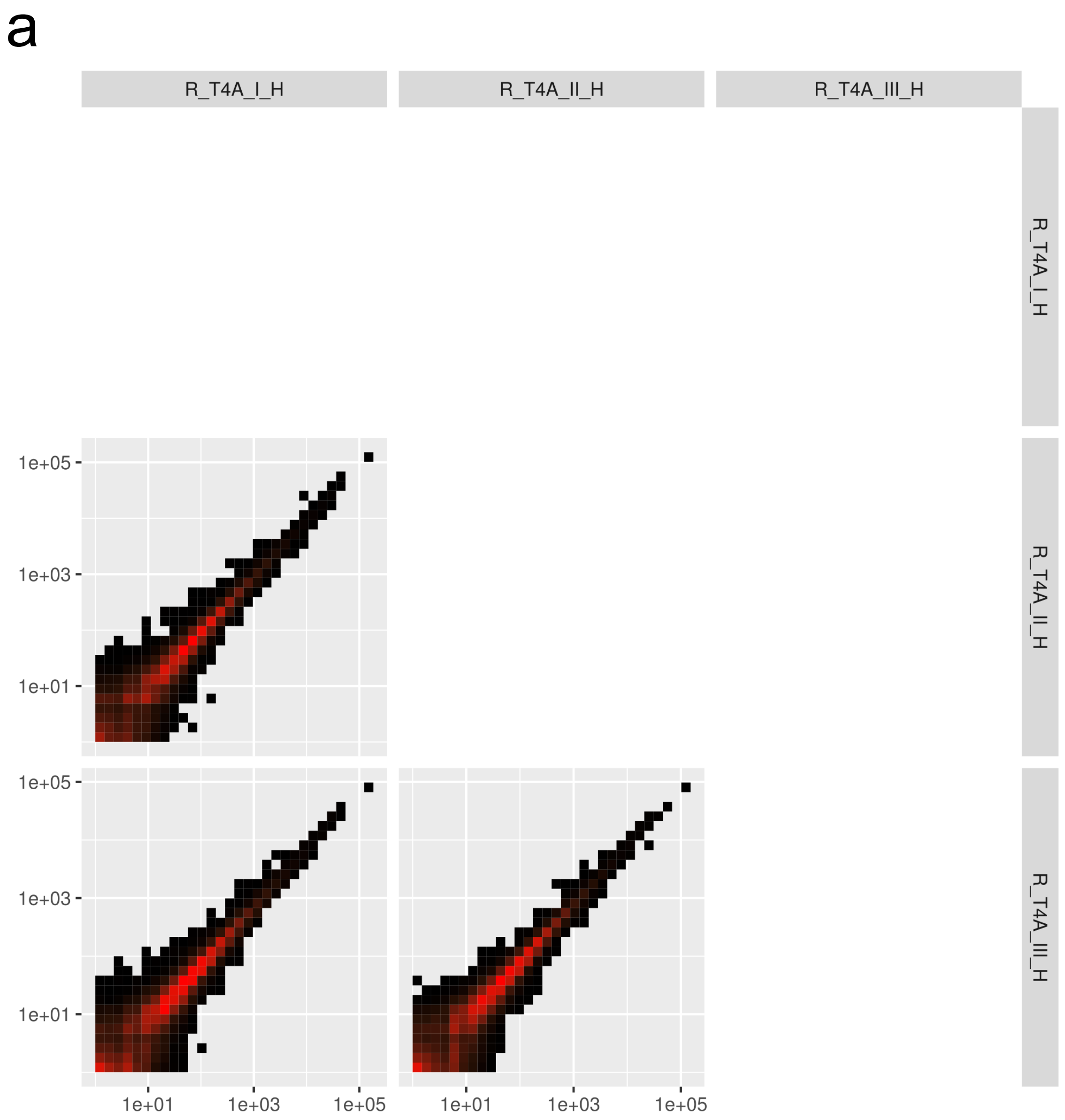

Additional file 3: Figure S1. Inter-replicate correlation plots

Inter-replicate correlation of genotype (a) PRA-A (non-symptomatic needles: NS), (b) PRA-A (symptomatic needles: S), (c) genotype PRA-B (NS), (d) PRA-B (S), (e) genotype PRA-D (NS), (f) PRA-D (S), (g) genotype PRA-R (NS), (h) PRA-R (S), (i) Pearson correlation coefficients.

b

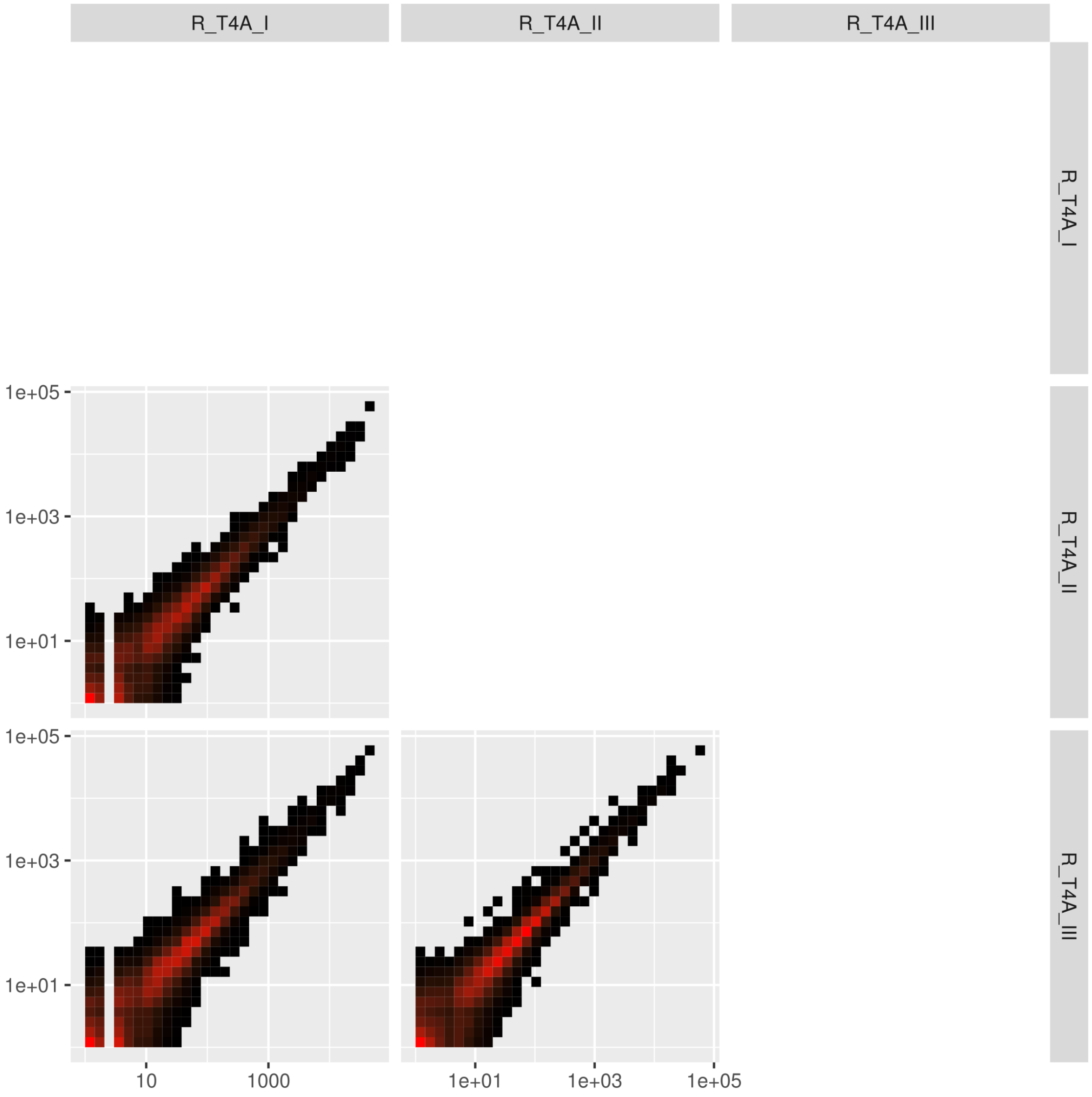
R_T4A_I
R_T4A_II
R_T4A_III
R_T4A_I
R_T4A_II
R_T4A_III
1e+05
1e+03
1e+01
1e+05
1e+03
1e+01
10
1000
1e+01
1e+03
1e+05

C

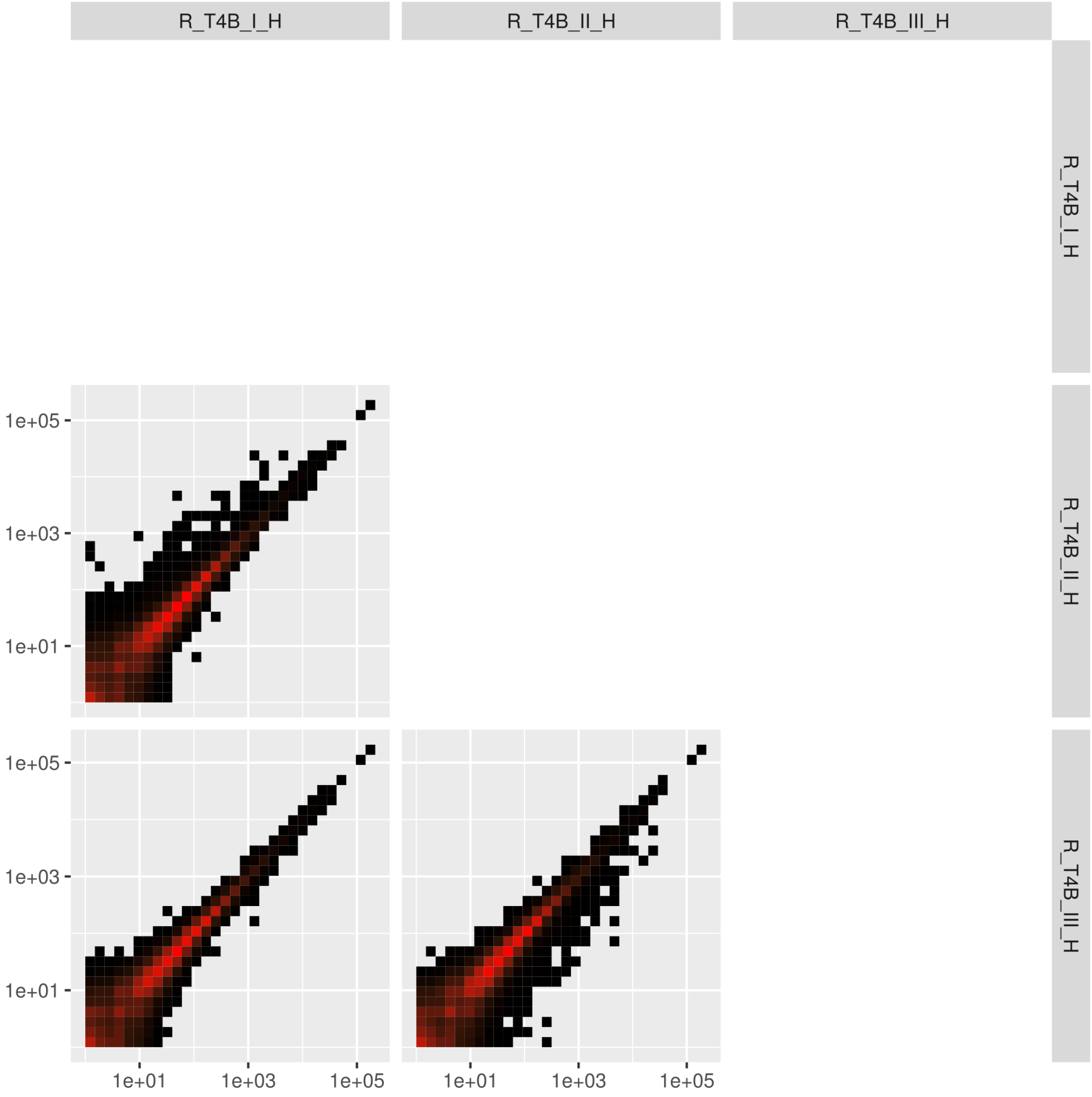

R_T4B_I_H
R_T4B_II_H
R_T4B_III_H
R_T4B_I_H
R_T4B_II_H
R_T4B_III_H
1e+05
1e+03
1e+01
1e+05
1e+03
1e+01
1e+01
1e+03
1e+05
1e+01
1e+03
1e+05

d

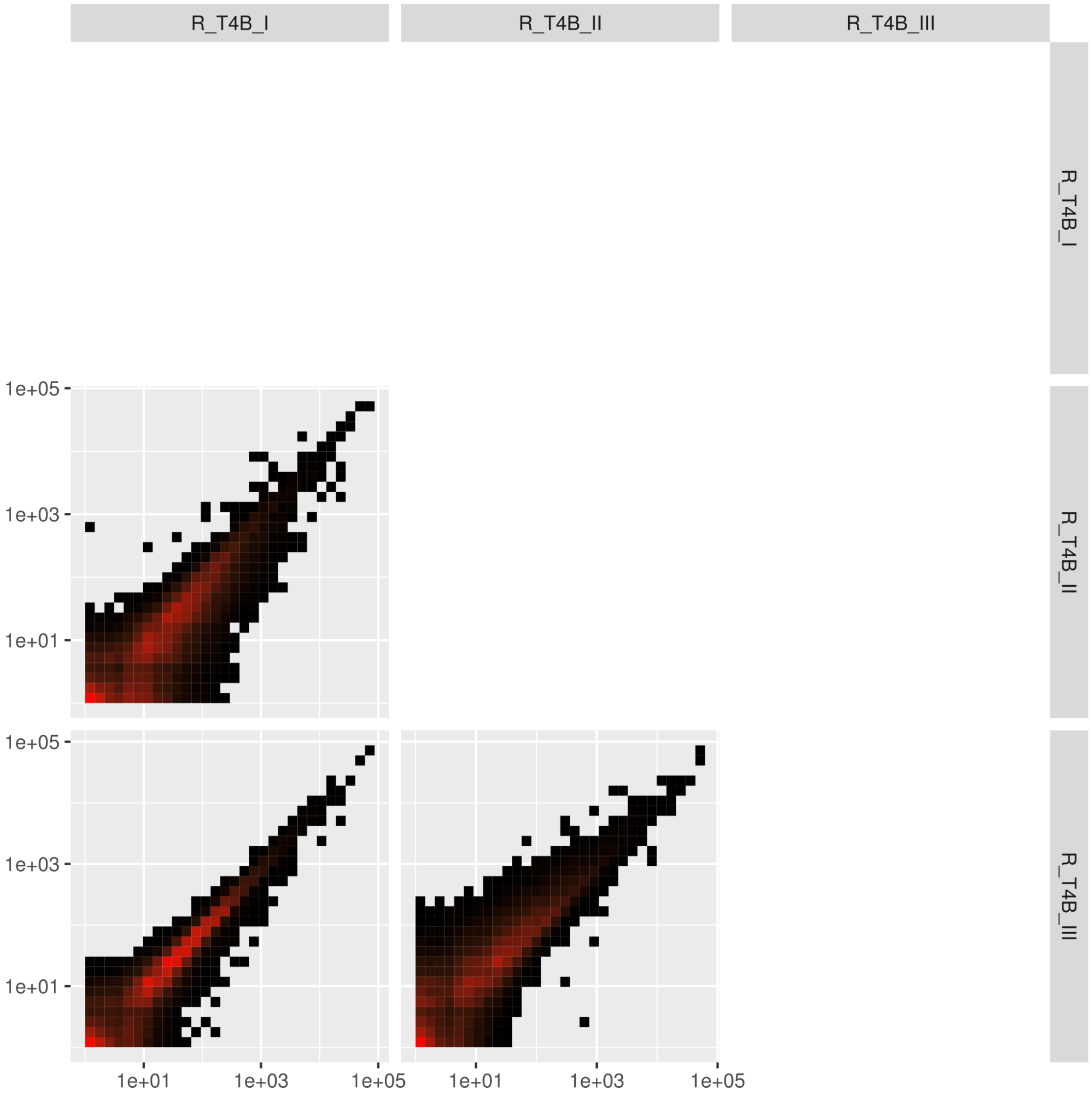

R_T4B_I
R_T4B_II
R_T4B_III
R_T4B_I
R_T4B_II
R_T4B_III
1e+05
1e+03
1e+01
1e+05
1e+03
1e+01
1e+01
1e+03
1e+05
1e+01
1e+03
1e+05

e

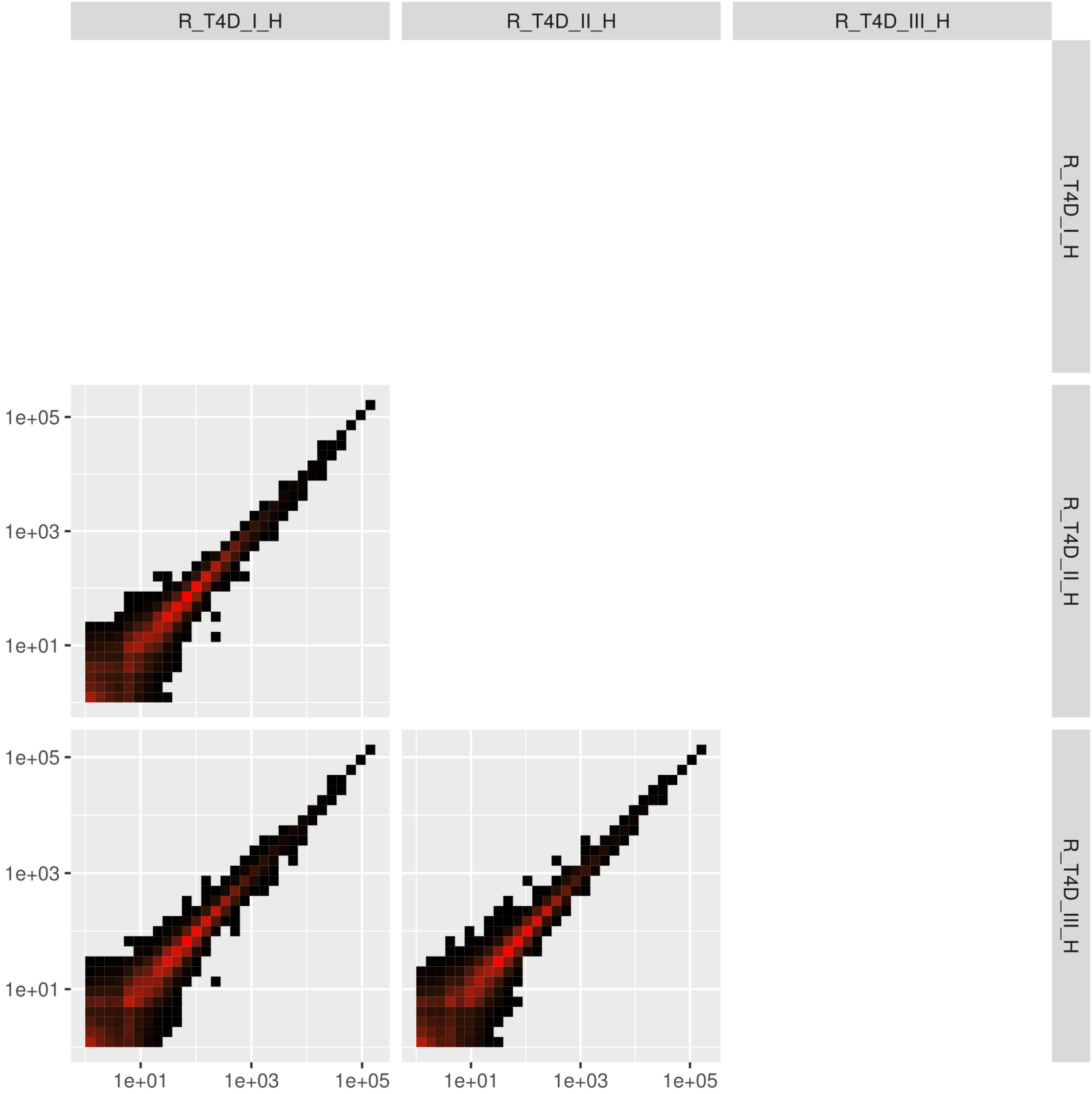
R_T4D_I_H
R_T4D_II_H
R_T4D_III_H
R_T4D_I_H
R_T4D_II_H
R_T4D_III_H
1e+05
1e+03
1e+01
1e+05
1e+03
1e+01
1e+01
1e+03
1e+05
1e+01
1e+03
1e+05

f

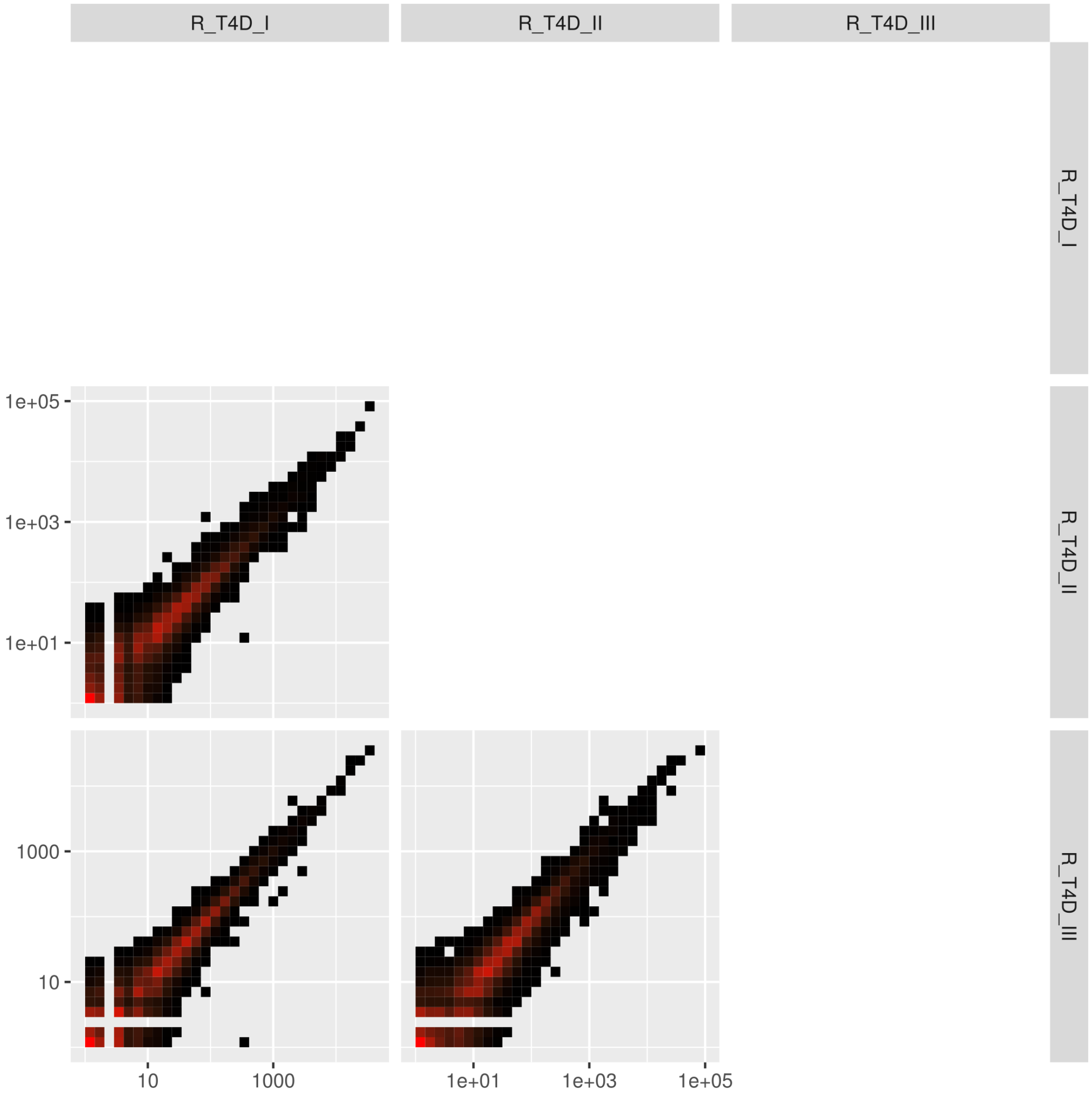

R_T4D_I
R_T4D_II
R_T4D_III
R_T4D_I
R_T4D_II
R_T4D_III
1e+05
1e+03
1e+01
1000
10
10
1000
1e+01
1e+03
1e+05

g

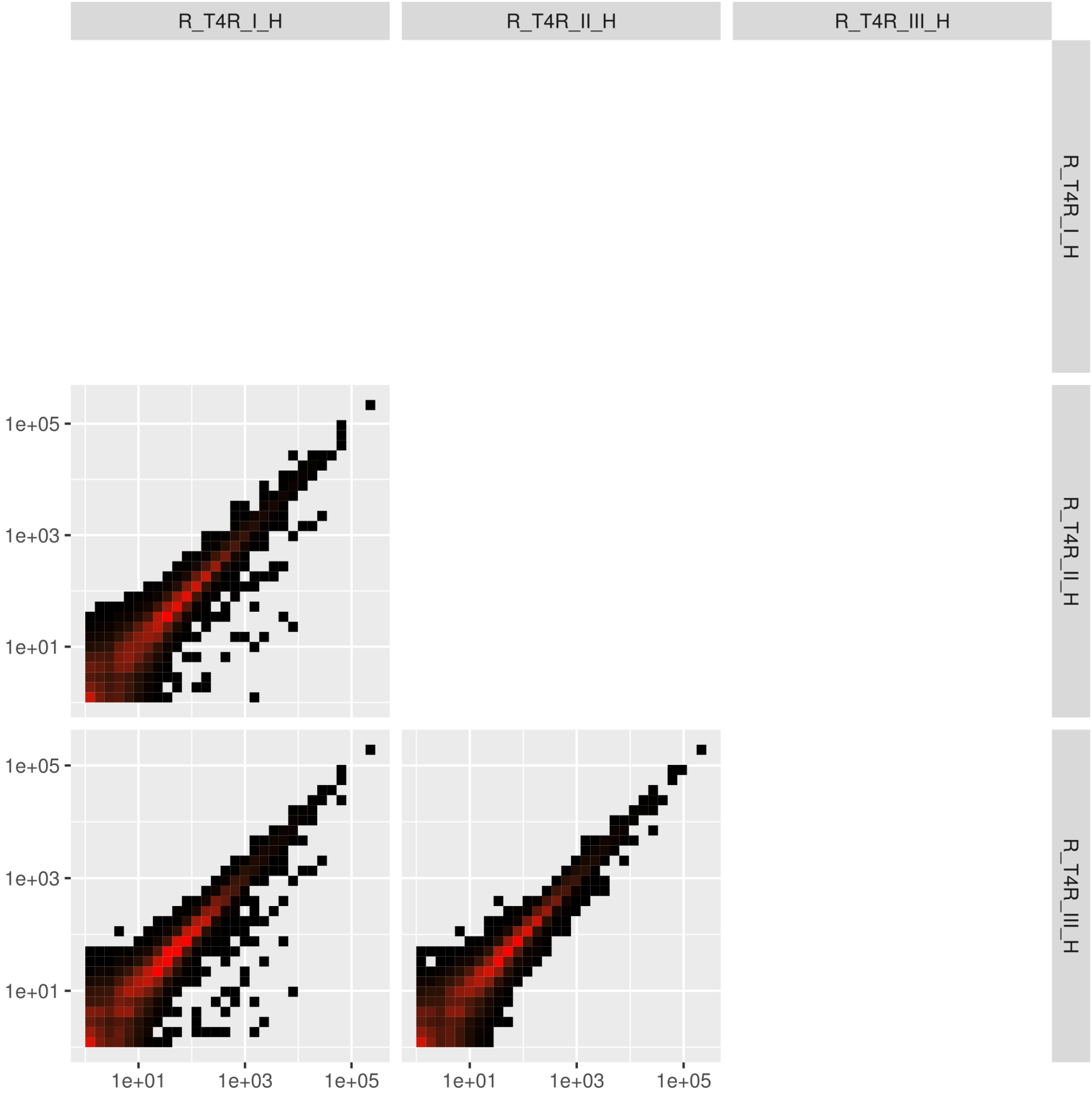
R_T4R_I_H
R_T4R_II_H
R_T4R_III_H
R_T4R_I_H
R_T4R_II_H
R_T4R_III_H
1e+05
1e+03
1e+01
1e+05
1e+03
1e+01
1e+01
1e+03
1e+05
1e+01
1e+03
1e+05

h

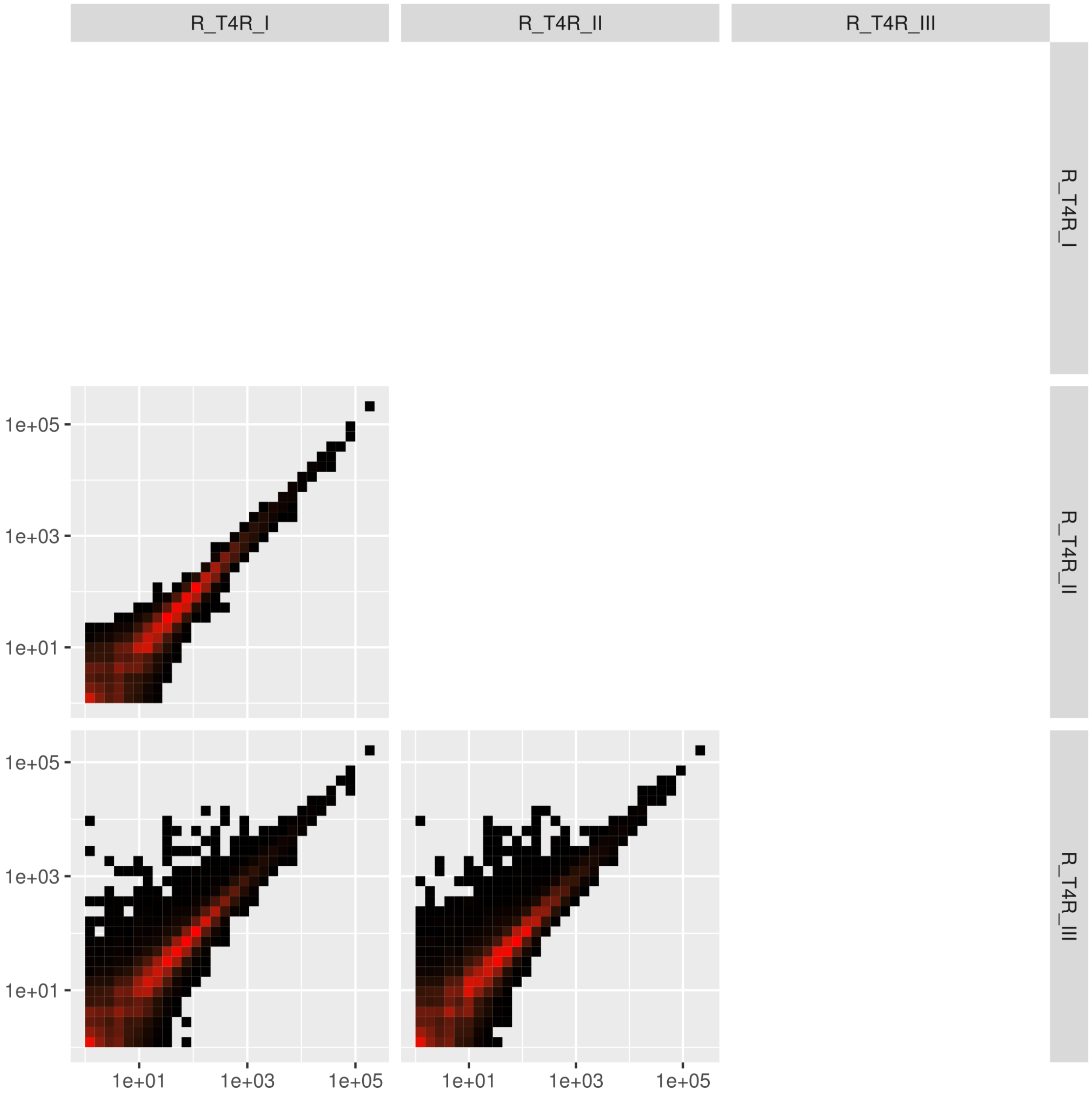
R_T4R_I
R_T4R_II
R_T4R_III
R_T4R_I
R_T4R_II
R_T4R_III
1e+05
1e+03
1e+01
1e+05
1e+03
1e+01
1e+01
1e+03
1e+05
1e+01
1e+03
1e+05

i

Sample1	Sample2	R_log
R_T4A_III_H	R_T4A_II_H	0.928488
R_T4A_III_H	R_T4A_I_H	0.921626
R_T4A_II_H	R_T4A_I_H	0.928031
R_T4A_I	R_T4A_II	0.915805
R_T4A_I	R_T4A_III	0.912889
R_T4A_II	R_T4A_III	0.925661
R_T4B_III_H	R_T4B_II_H	0.923085
R_T4B_III_H	R_T4B_I_H	0.93063
R_T4B_II_H	R_T4B_I_H	0.917963
R_T4B_I	R_T4B_II	0.828263
R_T4B_I	R_T4B_III	0.921584
R_T4B_II	R_T4B_III	0.834496
R_T4D_III_H	R_T4D_II_H	0.931706
R_T4D_III_H	R_T4D_I_H	0.931309
R_T4D_II_H	R_T4D_I_H	0.937067
R_T4D_I	R_T4D_II	0.910893
R_T4D_I	R_T4D_III	0.921862
R_T4D_II	R_T4D_III	0.908601
R_T4R_III_H	R_T4R_II_H	0.93453
R_T4R_III_H	R_T4R_I_H	0.926175
R_T4R_II_H	R_T4R_I_H	0.926612
R_T4R_I	R_T4R_II	0.936759
R_T4R_I	R_T4R_III	0.905187
R_T4R_II	R_T4R_III	0.900623
