# Supplementary material for: Advances in understanding Norway spruce natural resistance to needle bladder rust infection: transcriptional and secondary metabolites profiling
Source: BMC Genomics. 2022 Jun 13;23:435. doi: 10.1186/s12864-022-08661-y (PMC9190139; doi:10.1186/s12864-022-08661-y)
Supplement: Supplementary file 5 — Additional file 5: Figure S2. Numbers and proportion of differentially expressed genes. [file 12864_2022_8661_MOESM5_ESM.docx]

**Additional file 5: Figure S2.** **Numbers and proportion of differentially expressed genes.**

(a) Absolute numbers (b) and proportions of over- and under-expressed genes of symptomatic (S) vs. non-symptomatic needles (NS) for all genotypes (PRA-R, PRA-A, PRA-B and PRA-D) and the contrast between NS needles of PRA-R vs. NS needles of all susceptible genotypes (Rn vs. An, Rn vs. Bn, Rn vs. Dn).
