# Supplementary material for: Advances in understanding Norway spruce natural resistance to needle bladder rust infection: transcriptional and secondary metabolites profiling
Source: BMC Genomics. 2022 Jun 13;23:435. doi: 10.1186/s12864-022-08661-y (PMC9190139; doi:10.1186/s12864-022-08661-y)
Supplement: Supplementary file 6 — Additional file 6: Figure S3. Induced defence: Gene ontology (GO) term enrichment analysis [file 12864_2022_8661_MOESM6_ESM.pdf]

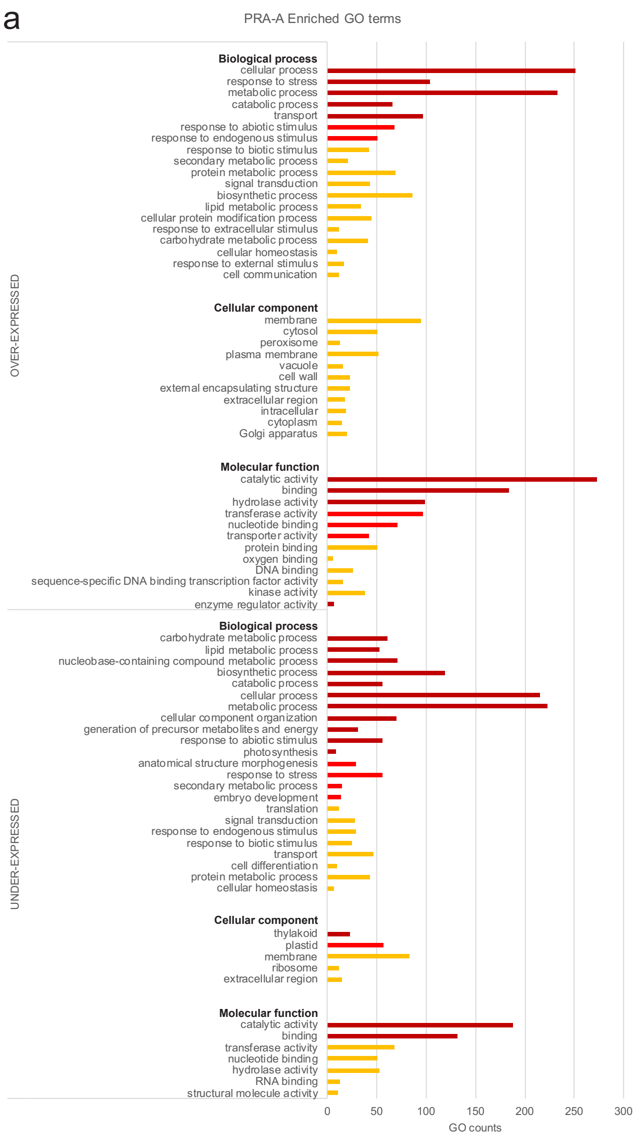

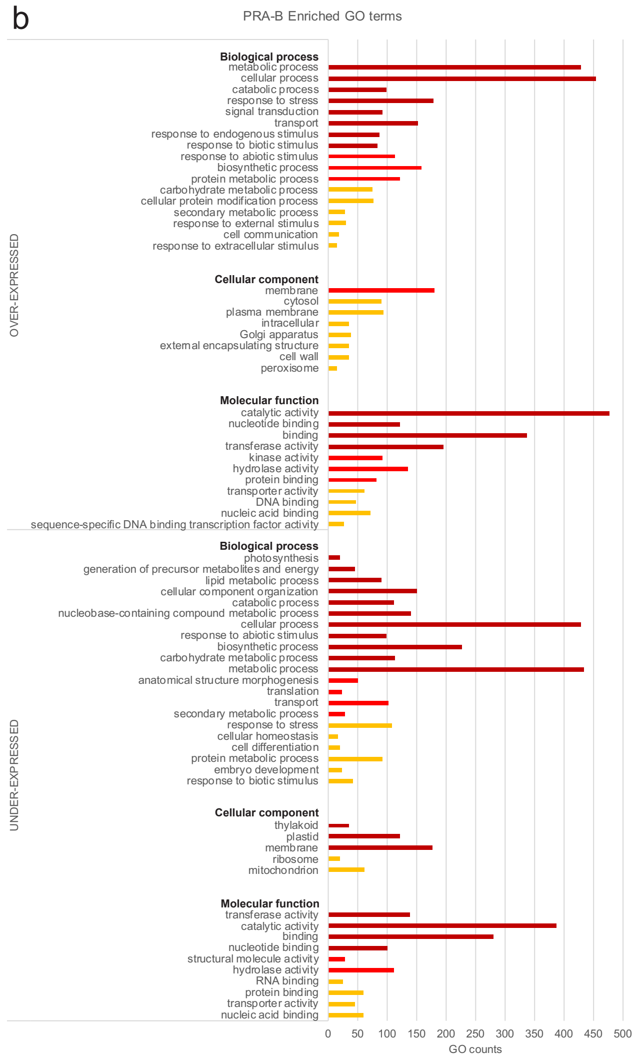

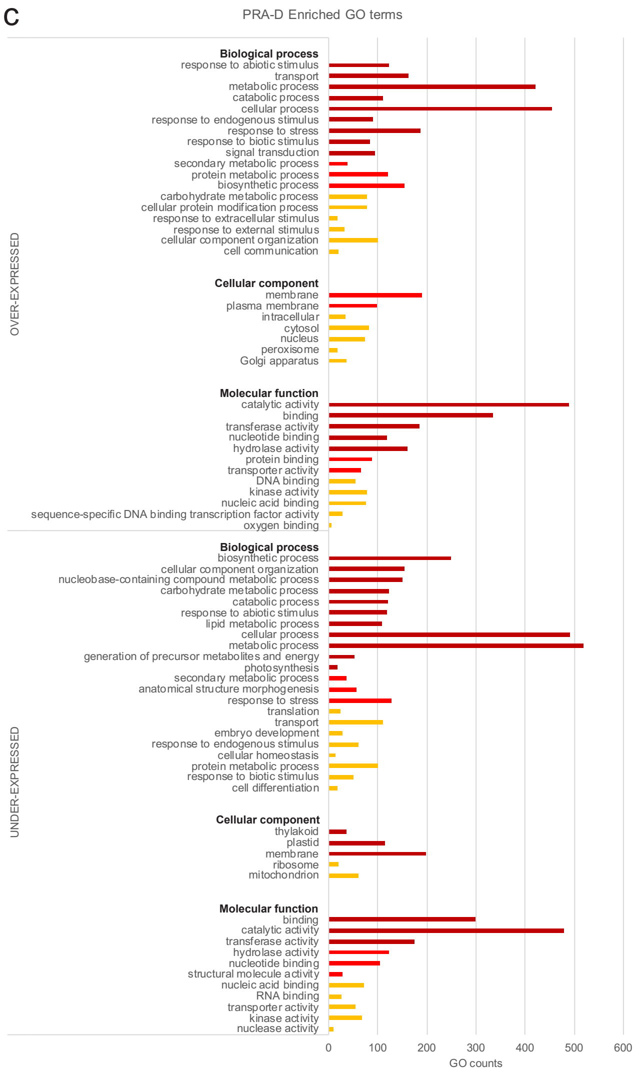

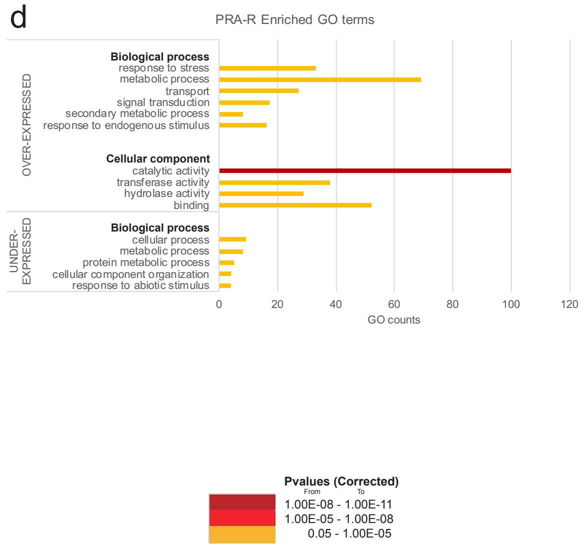

Additional file 6: Figure S3. Induced defence: Gene ontology (GO) term enrichment analysis

GO terms overrepresented in differentially over- or under-expressed transcripts for each genotype in symptomatic needles (S) compared to non-symptomatic needles (NS), once symptoms of infection by C. rhododendri were visible: (a) PRA-A, (b) PRA-B, (c) PRA-D, (d) PRA-R. Terms were ranked by the corrected p-value.
