# Supplementary material for: Advances in understanding Norway spruce natural resistance to needle bladder rust infection: transcriptional and secondary metabolites profiling
Source: BMC Genomics. 2022 Jun 13;23:435. doi: 10.1186/s12864-022-08661-y (PMC9190139; doi:10.1186/s12864-022-08661-y)

Additional file 11: Figure S5. Induced defence: Heat map plot for DEGs belonging to defence pathways

Comparative pathway analysis of susceptible genotypes analysed in this study with the genotype ASS-7 (reported in Trujillo-Moya et al. 2020). The results revealed the similarity of all susceptible genotypes samples collected 38 days post infection (dpi) with the 39 dpi ASS-7 samples (better than 21 dpi ASS-7 samples) for both over- and under-expressed DEGs subsets. For ASS-7, two types of pathway fingerprints were included derived from the following contrasts: symptomatic (S) needles from infected plant (controlled artificial infection) vs. needles from control (C) not infected plant (S-C); S needles from infected plant vs. non-symptomatic (NS) needles from the same infected plant (S-NS). Columns (samples) and rows (genes) were sorted by clustering (clusters data by similarity), Euclidean distance was used as distance metric. Log2fold change represented at each gene is shown from green (-3.14) to red colour (7.65). All genes (331) belonging to the ten defence pathways explored before by (Trujillo-Moya et al. 2020) were included here.

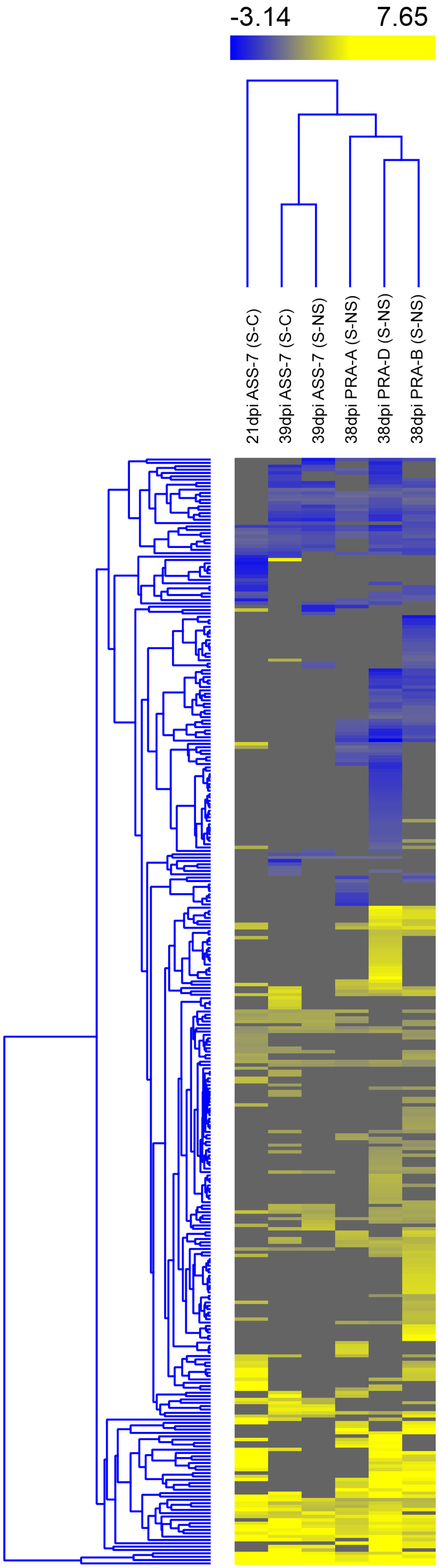

Supplement: Supplementary file 11 — Additional file 11: Figure S5. Induced defence: Heat map plot for DEGs belonging to defence pathways. [file 12864_2022_8661_MOESM11_ESM.pdf]
