# Supplementary material for: Advances in understanding Norway spruce natural resistance to needle bladder rust infection: transcriptional and secondary metabolites profiling
Source: BMC Genomics. 2022 Jun 13;23:435. doi: 10.1186/s12864-022-08661-y (PMC9190139; doi:10.1186/s12864-022-08661-y)
Supplement: Supplementary file 13 — Additional file 13: Figure S6. Gene ontology (GO) term enrichment analysis for DEGs in PRA-R non-symptomatic needles (NS) contrasted to NS needles from all susceptible genotypes. [file 12864_2022_8661_MOESM13_ESM.pdf]

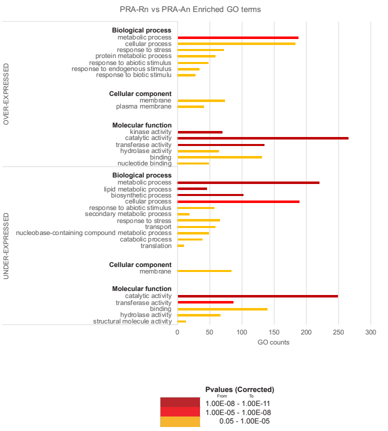

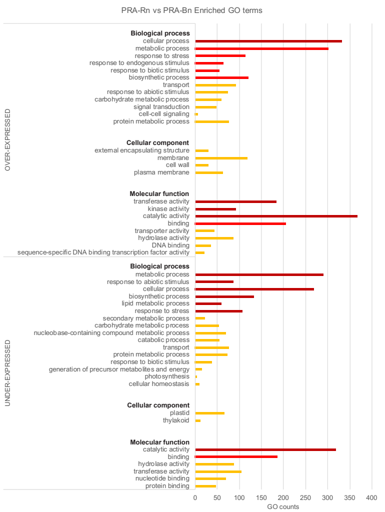

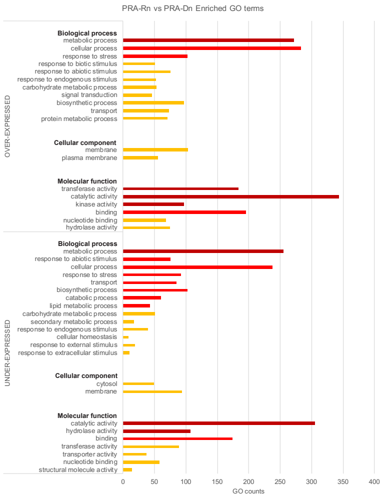

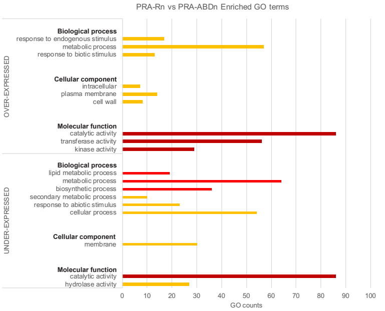

Additional file 13: Figure S6. Gene ontology (GO) term enrichment analysis for DEGs in PRA-R non-symptomatic needles (NS) contrasted to NS needles from all susceptible genotypes

GO terms overrepresented in over- or under-expressed transcripts differentially expressed for the contrast between PRA-R NS needles compared to NS needles from all susceptible genotypes, once symptoms of infection were visible. Additionally, DEGs shared by all contrasts between PRA-R NS (PRA-Rn) vs. PRA-A/B/D NS needles (PRA-A/B/Dn) was included. Terms ranked by the corrected p-value.
