# Supplementary material for: Advances in understanding Norway spruce natural resistance to needle bladder rust infection: transcriptional and secondary metabolites profiling
Source: BMC Genomics. 2022 Jun 13;23:435. doi: 10.1186/s12864-022-08661-y (PMC9190139; doi:10.1186/s12864-022-08661-y)
Supplement: Supplementary file 15 — Additional file 15: Figure S7. Constitutive differences between the resistant and susceptible genotypes viewed through heat maps of DEGs distribution across pathways (pathway fingerprint). [file 12864_2022_8661_MOESM15_ESM.docx]

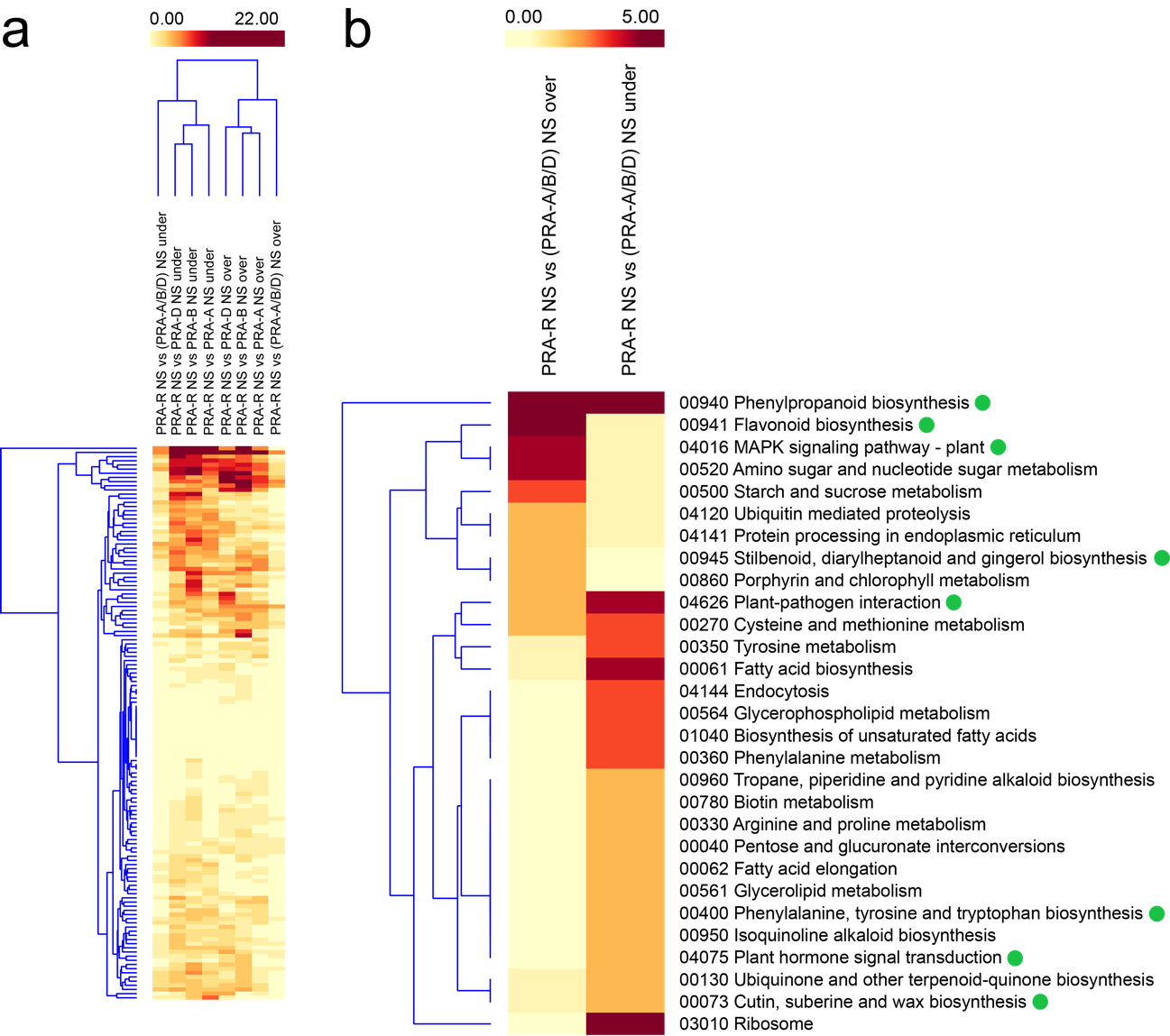


**Additional file 15: Figure S7. Constitutive differences between the resistant and susceptible genotypes viewed through heat maps of DEGs distribution across pathways (pathway fingerprint).**

(a) DEGs distribution across pathways for all independent contrasts between PRA-R NS needles and susceptible genotypes NS needles (PRA-R NS vs. PRA-A NS, PRA-R NS vs. PRA-B NS and PRA-R NS vs. PRA-D NS), grouped together with the subset of 706 shared transcripts (PRA-R NS vs. PRA-A/B/C NS) for both under and over-expressed DEGs. (b) Among the shared DEGs, we found a set of DEGs coding for enzymes previously proposed to be involved in plant defence pathways (Trujillo-Moya et al. 2020), marked with a green dot. The number of enzymes-encoding genes found within each pathway is indicated using a colour scale from light yellow (0) to dark red colour (22 for “a” and 5 for “b”).
