# Supplementary material for: Advances in understanding Norway spruce natural resistance to needle bladder rust infection: transcriptional and secondary metabolites profiling
Source: BMC Genomics. 2022 Jun 13;23:435. doi: 10.1186/s12864-022-08661-y (PMC9190139; doi:10.1186/s12864-022-08661-y)
Supplement: Supplementary file 18 — Additional file 18: Figure S8. RT-qPCR results of selected differentially expressed transcripts. [file 12864_2022_8661_MOESM18_ESM.pdf]

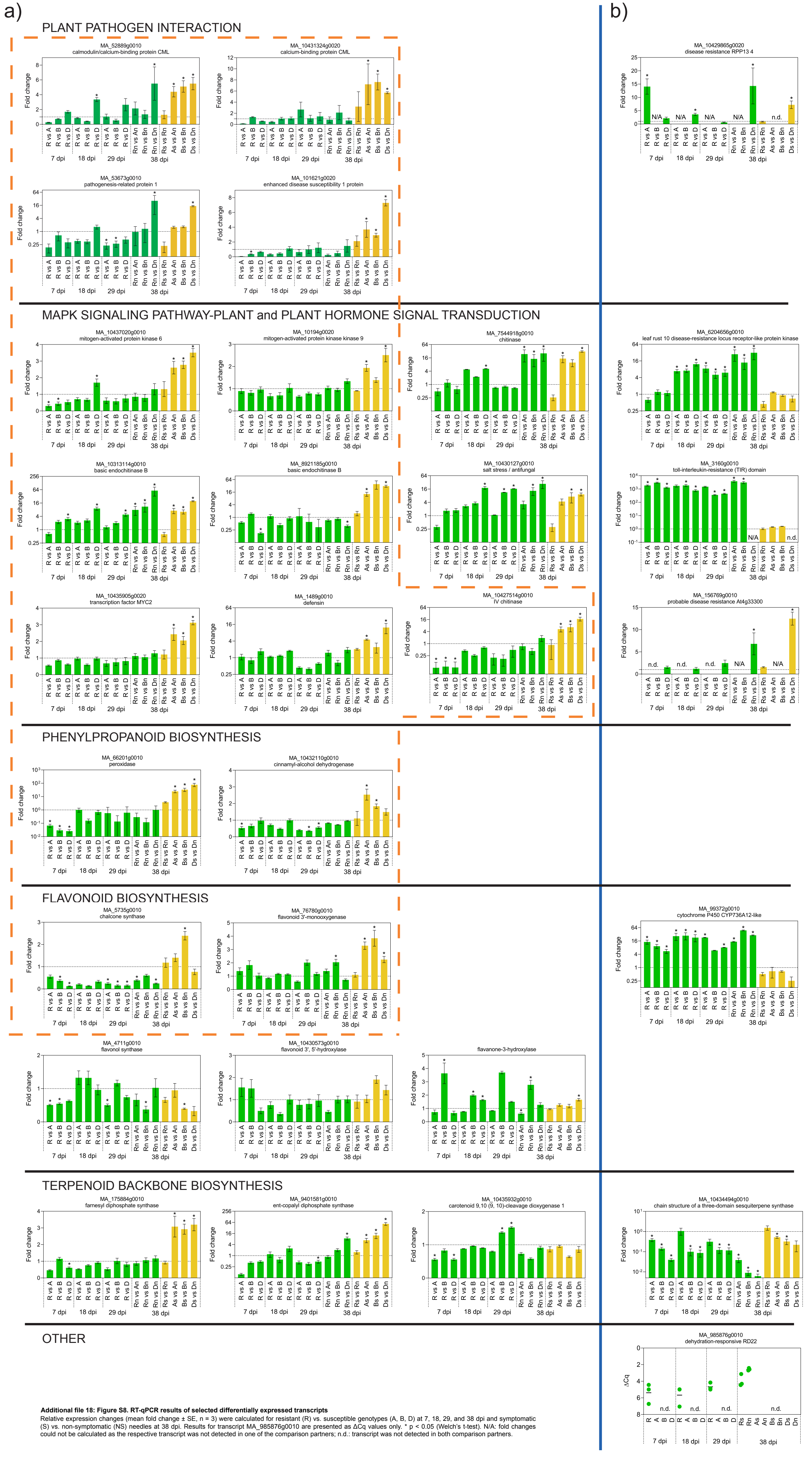

Additional file 18: Figure S8. RT-qPCR results of selected differentially expressed transcripts

Relative expression changes (mean fold change ± SE, n = 3) were calculated for resistant (R) vs. susceptible genotypes (A, B, D) at 7, 18, 29, and 38 dpi and symptomatic (S) vs. non-symptomatic (NS) needles at 38 dpi. Results for transcript MA_985876g0010 are presented as ΔCq values only. * $p < 0.05$ (Welch's t-test). N/A: fold changes could not be calculated as the respective transcript was not detected in one of the comparison partners; n.d.: transcript was not detected in both comparison partners.
