# Supplementary material for: Advances in understanding Norway spruce natural resistance to needle bladder rust infection: transcriptional and secondary metabolites profiling
Source: BMC Genomics. 2022 Jun 13;23:435. doi: 10.1186/s12864-022-08661-y (PMC9190139; doi:10.1186/s12864-022-08661-y)
Supplement: Supplementary file 22 — Additional file 22: Figure S9. Principal component analysis (PCA) of terpene contents over time. [file 12864_2022_8661_MOESM22_ESM.docx]

**Additional file 22: Figure S9.** **Principal component analysis (PCA) of terpene contents over time.**

Note how the first component (PC1) separates the resistant genotype PRA-R from the susceptible genotypes PRA-A, PRA-B and PRA-D at 7 dpi, and how PRA-A becomes more similar to PRA-R with time.
