# Supplementary material for: Advances in understanding Norway spruce natural resistance to needle bladder rust infection: transcriptional and secondary metabolites profiling
Source: BMC Genomics. 2022 Jun 13;23:435. doi: 10.1186/s12864-022-08661-y (PMC9190139; doi:10.1186/s12864-022-08661-y)
Supplement: Supplementary file 23 — Additional file 23: Figure S10. Terpenes with different content levels in the resistant genotype PRA-R compared to susceptible genotypes at all time points. [file 12864_2022_8661_MOESM23_ESM.pdf]

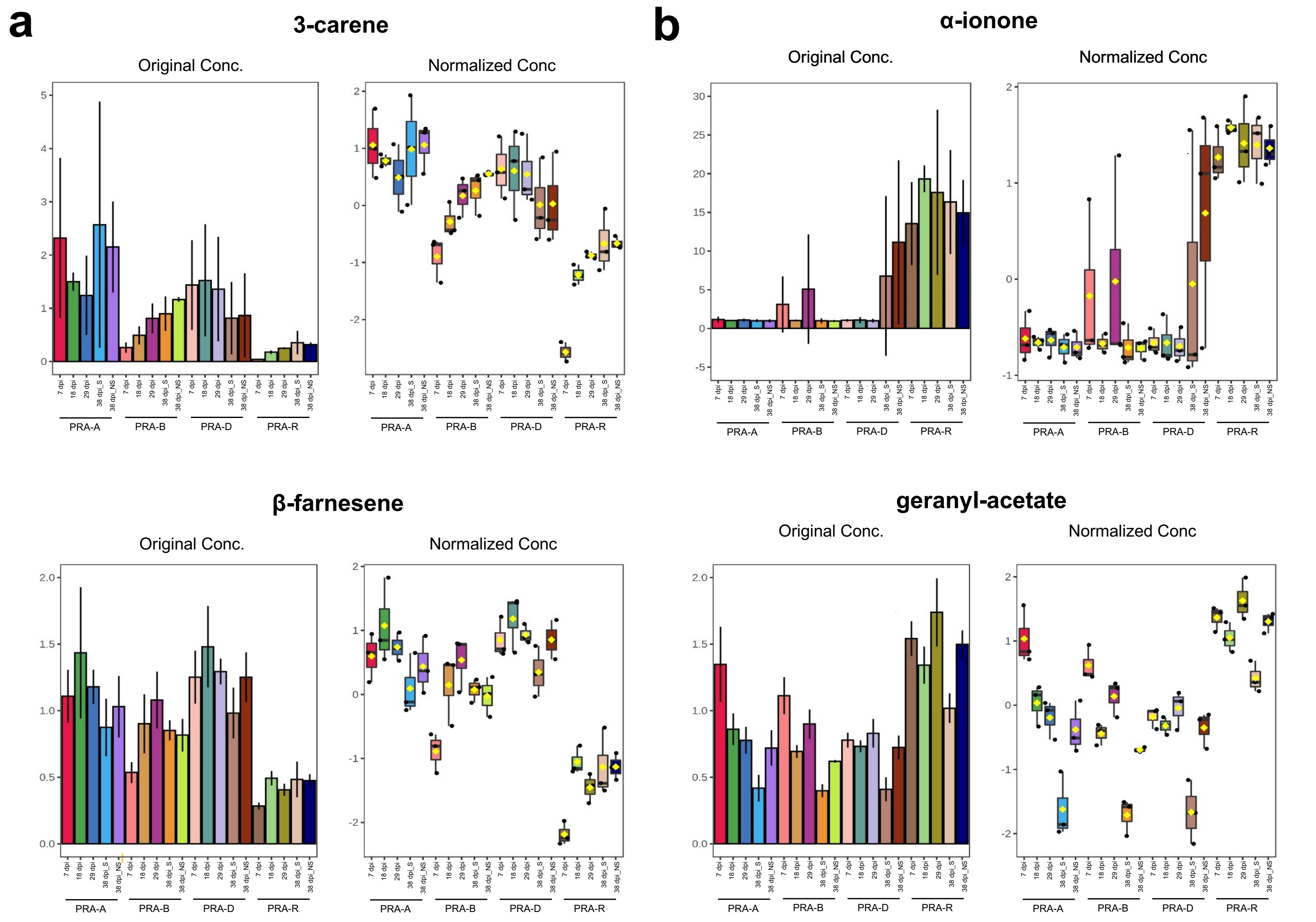

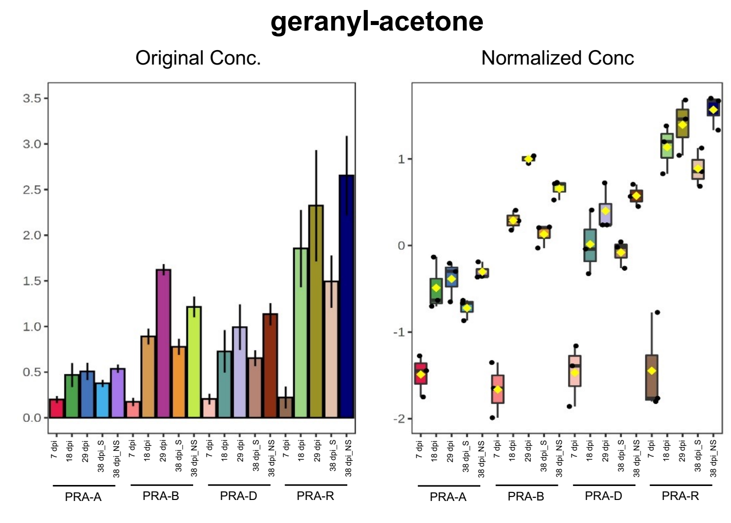

Additional file 23: Figure S10. Terpenes with different content levels in the resistant genotype PRA-R compared to susceptible genotypes at all time points
(a) 3-carene and β-farnesene were consistently lower in PRA-R at all time points and (b) geranyl acetone, α-ionone and geranyl acetate were consistently higher in PRA-R at all time points.
