# Supplementary material for: Advances in understanding Norway spruce natural resistance to needle bladder rust infection: transcriptional and secondary metabolites profiling
Source: BMC Genomics. 2022 Jun 13;23:435. doi: 10.1186/s12864-022-08661-y (PMC9190139; doi:10.1186/s12864-022-08661-y)
Supplement: Supplementary file 24 — Additional file 24: Figure S11. Content levels of all individual phenolic compounds. [file 12864_2022_8661_MOESM24_ESM.docx]

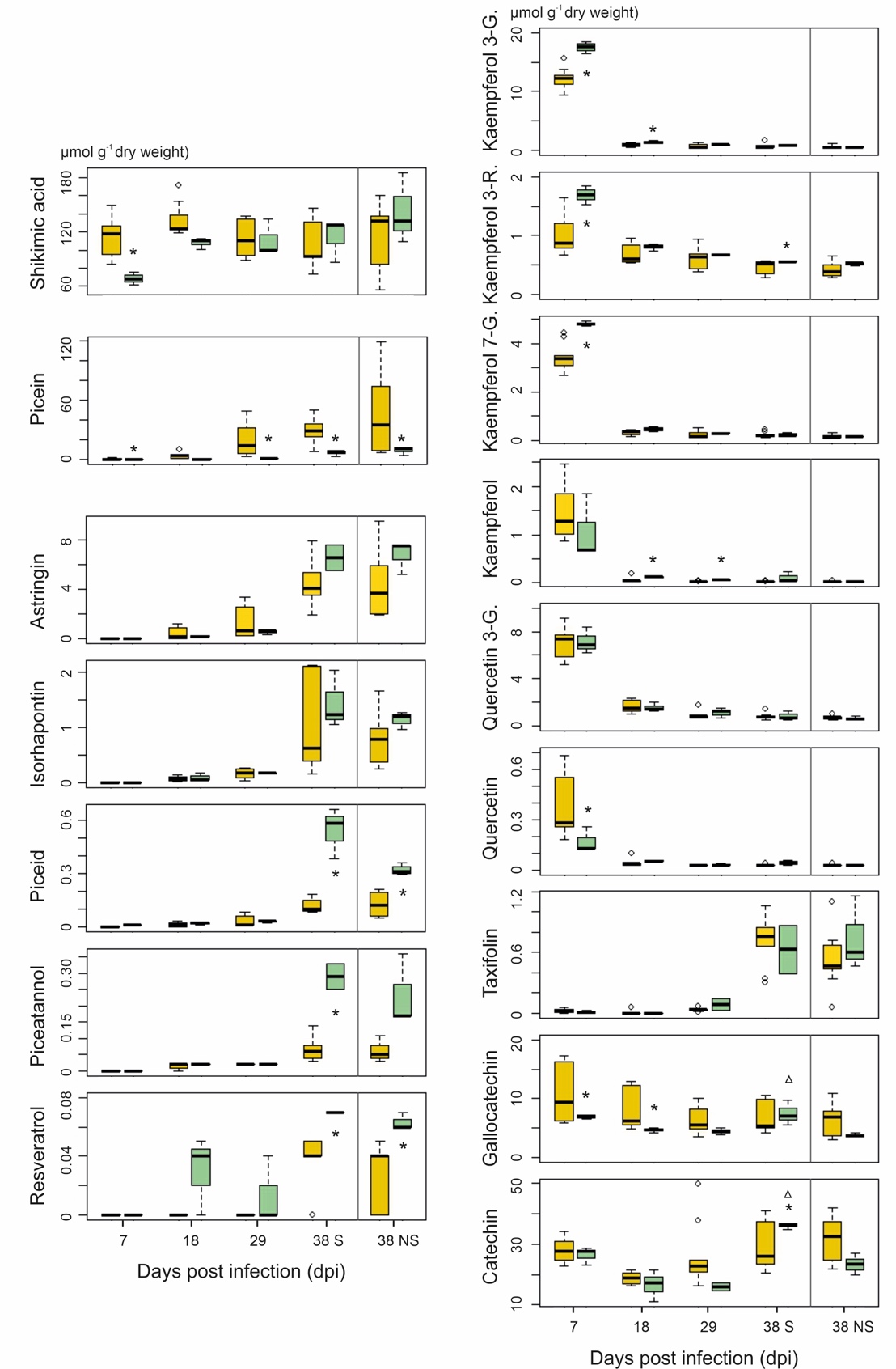


**Additional file 24: Figure S11.** **Content levels of all individual phenolic compounds.**

Phenolic compound contents between 7 and 38 dpi in needles of susceptible genotypes (pooled data of PRA-A/B/D; yellow boxes) and the resistant genotype PRA-R (green boxes). For 38 dpi, results for both symptomatic (S) and non-symptomatic (NS) needles are shown. Three branches per tree were analysed; significantly different contents in PRA-R compared to susceptible genotypes are marked with asterisks and in 38 dpi S compared to NS needles with a triangle.
