# Supplementary material for: Advances in understanding Norway spruce natural resistance to needle bladder rust infection: transcriptional and secondary metabolites profiling
Source: BMC Genomics. 2022 Jun 13;23:435. doi: 10.1186/s12864-022-08661-y (PMC9190139; doi:10.1186/s12864-022-08661-y)
Supplement: Supplementary file 25 — Additional file 25: Table S13. Raw data LC–MS analysis. [file 12864_2022_8661_MOESM25_ESM.pdf]

Additional file 25: Table S13. Raw data LC-MS analysis

Needle content of all analysed phenolic compounds and plant hormones for each genotype and time point. Mean ± standard error of three measurements.

		precursor	*simple phenylprop.*	*stilbene*	*stilbene*	*stilbene*	*stilbene*	*stilbene*	*total stilbenes*	*flavonoid*	*flavonoid*
genotype	dpi	shikimic acid	picein	astringin	isorhapontin	piceid	piceatannol	resveratrol		kaempferol	kaempferol 3-glucoside
PRA-R	7	68.46 ± 4.35	0.12 ± 0.03	0.03 ± 0.00	0.00 ± 0.00	0.01 ± 0.00	0.00 ± 0.00	0.00 ± 0.00	0.04 ± 0.00	1.07 ± 0.39	17.53 ± 0.60
PRA-R	18	108.12 ± 3.64	0.49 ± 0.16	0.21 ± 0.04	0.09 ± 0.05	0.02 ± 0.00	0.02 ± 0.00	0.03 ± 0.01	0.36 ± 0.09	0.13 ± 0.01	1.40 ± 0.15
PRA-R	29	110.75 ± 11.60	1.28 ± 0.29	0.54 ± 0.10	0.17 ± 0.01	0.03 ± 0.00	0.02 ± 0.00	0.01 ± 0.01	0.71 ± 0.06	0.06 ± 0.01	0.97 ± 0.12
PRA-R	38 S	113.99 ± 13.95	6.39 ± 1.66	6.56 ± 0.84	1.44 ± 0.30	0.54 ± 0.08	0.29 ± 0.03	0.07 ± 0.00	8.54 ± 0.69	0.12 ± 0.06	0.85 ± 0.11
PRA-R	38 NS	142.17 ± 22.48	9.15 ± 2.37	6.74 ± 0.75	1.14 ± 0.09	0.32 ± 0.02	0.23 ± 0.06	0.06 ± 0.01	8.49 ± 0.68	0.04 ± 0.00	0.59 ± 0.04
PRA-A	7	100.44 ± 12.62	0.24 ± 0.03	0.03 ± 0.00	0.00 ± 0.00	0.00 ± 0.00	0.00 ± 0.00	0.00 ± 0.00	0.04 ± 0.00	1.65 ± 0.32	13.67 ± 1.16
PRA-A	18	123.85 ± 0.57	4.97 ± 2.68	0.36 ± 0.27	0.04 ± 0.02	0.01 ± 0.01	0.01 ± 0.01	0.00 ± 0.00	0.42 ± 0.30	0.10 ± 0.05	1.16 ± 0.12
PRA-A	29	112.59 ± 11.56	14.38 ± 4.97	0.67 ± 0.25	0.07 ± 0.02	0.02 ± 0.01	0.02 ± 0.00	0.00 ± 0.00	0.77 ± 0.27	0.03 ± 0.00	1.12 ± 0.14
PRA-A	38 S	142.17 ± 22.48	9.15 ± 2.37	6.74 ± 0.75	1.14 ± 0.09	0.32 ± 0.02	0.23 ± 0.06	0.06 ± 0.01	2.92 ± 0.41	0.04 ± 0.00	0.59 ± 0.04
PRA-A	38 NS	103.37 ± 10.73	19.56 ± 6.13	2.56 ± 0.51	0.23 ± 0.05	0.09 ± 0.01	0.03 ± 0.00	0.01 ± 0.01	4.38 ± 0.41	0.04 ± 0.01	1.40 ± 0.30
PRA-B	7	103.55 ± 7.32	0.45 ± 0.04	0.03 ± 0.00	0.01 ± 0.00	0.00 ± 0.00	0.00 ± 0.00	0.00 ± 0.00	0.04 ± 0.00	1.73 ± 0.47	10.84 ± 0.82
PRA-B	18	119.84 ± 1.01	1.59 ± 0.35	0.11 ± 0.06	0.05 ± 0.00	0.00 ± 0.00	0.01 ± 0.01	0.00 ± 0.00	0.15 ± 0.04	0.05 ± 0.00	1.01 ± 0.08
PRA-B	29	96.15 ± 5.80	5.13 ± 2.04	0.34 ± 0.07	0.21 ± 0.03	0.01 ± 0.00	0.02 ± 0.00	0.00 ± 0.00	0.58 ± 0.10	0.04 ± 0.01	0.69 ± 0.22
PRA-B	38 S	103.37 ± 10.73	19.56 ± 6.13	2.56 ± 0.51	0.23 ± 0.05	0.09 ± 0.01	0.03 ± 0.00	0.01 ± 0.01	6.45 ± 0.23	0.04 ± 0.01	1.40 ± 0.30
PRA-B	38 NS	141.16 ± 9.74	44.85 ± 12.24	3.91 ± 0.38	0.32 ± 0.04	0.12 ± 0.01	0.03 ± 0.00	0.00 ± 0.00	3.00 ± 0.11	0.03 ± 0.00	0.84 ± 0.16
PRA-D	7	136.09 ± 7.03	1.38 ± 0.12	0.07 ± 0.00	0.00 ± 0.00	0.00 ± 0.00	0.00 ± 0.00	0.00 ± 0.00	0.08 ± 0.00	0.99 ± 0.04	12.07 ± 0.43
PRA-D	18	148.73 ± 14.09	4.06 ± 0.26	0.96 ± 0.16	0.12 ± 0.01	0.03 ± 0.01	0.02 ± 0.00	0.00 ± 0.00	1.13 ± 0.17	0.03 ± 0.00	0.62 ± 0.05
PRA-D	29	130.61 ± 5.50	39.32 ± 5.14	3.04 ± 0.25	0.21 ± 0.03	0.07 ± 0.01	0.02 ± 0.00	0.00 ± 0.00	3.34 ± 0.29	0.03 ± 0.00	0.38 ± 0.04
PRA-D	38 S	141.16 ± 9.74	44.85 ± 12.24	3.91 ± 0.38	0.32 ± 0.04	0.12 ± 0.01	0.03 ± 0.00	0.00 ± 0.00	6.93 ± 1.18	0.03 ± 0.00	0.84 ± 0.16
PRA-D	38 NS	137.44 ± 4.49	32.07 ± 1.83	4.14 ± 0.23	2.11 ± 0.01	0.09 ± 0.00	0.06 ± 0.01	0.04 ± 0.00	9.91 ± 0.70	0.03 ± 0.00	0.43 ± 0.03

		flavonoid	*flavonoid*	*flavonoid*	*flavonoid*	*flavonoid*	*flavonoid*	*flavonoid*	*total flavonoids*	*hormone*	*hormone*
genotype	dpi	kaempferol 3-rutinoside	kaempferol 7-glucoside	quercetin	quercetin 3-glucoside	taxifolin	gallocatechin	catechin		abscisic acid	salicylic acid
PRA-R	7	1.69 ± 0.09	4.81 ± 0.06	0.17 ± 0.04	7.15 ± 0.64	0.02 ± 0.01	6.92 ± 0.24	26.32 ± 1.69	65.68 ± 0.91	0.010 ± 0.001	0.006 ± 0.001
PRA-R	18	0.80 ± 0.03	0.47 ± 0.06	0.06 ± 0.00	1.55 ± 0.23	0.00 ± 0.00	4.64 ± 0.21	16.59 ± 2.95	25.63 ± 3.46	0.015 ± 0.002	0.014 ± 0.003
PRA-R	29	0.67 ± 0.02	0.28 ± 0.02	0.04 ± 0.00	1.11 ± 0.25	0.09 ± 0.05	4.44 ± 0.35	15.95 ± 1.05	18.03 ± 5.60	0.008 ± 0.001	0.024 ± 0.005
PRA-R	38 S	0.56 ± 0.01	0.25 ± 0.04	0.04 ± 0.01	0.82 ± 0.24	0.63 ± 0.19	7.43 ± 1.21	35.99 ± 0.61	46.48 ± 1.81	0.007 ± 0.001	0.171 ± 0.009
PRA-R	38 NS	0.52 ± 0.01	0.17 ± 0.01	0.03 ± 0.00	0.62 ± 0.11	0.74 ± 0.21	3.74 ± 0.20	23.38 ± 2.08	29.82 ± 2.31	0.005 ± 0.001	0.145 ± 0.016
PRA-A	7	1.03 ± 0.31	3.93 ± 0.42	0.42 ± 0.13	6.48 ± 1.00	0.02 ± 0.01	16.87 ± 0.30	24.67 ± 0.26	68.73 ± 0.62	0.010 ± 0.002	0.006 ± 0.000
PRA-A	18	0.64 ± 0.05	0.39 ± 0.03	0.06 ± 0.02	1.44 ± 0.35	0.00 ± 0.00	12.59 ± 0.22	19.97 ± 0.79	36.35 ± 1.37	0.009 ± 0.003	0.011 ± 0.003
PRA-A	29	0.60 ± 0.07	0.37 ± 0.07	0.03 ± 0.00	0.71 ± 0.05	0.03 ± 0.01	8.94 ± 1.08	26.84 ± 5.61	38.68 ± 6.64	0.003 ± 0.001	0.014 ± 0.004
PRA-A	38 S	0.52 ± 0.01	0.17 ± 0.01	0.03 ± 0.00	0.62 ± 0.11	0.74 ± 0.21	3.74 ± 0.20	23.38 ± 2.08	53.38 ± 1.03	0.005 ± 0.001	0.145 ± 0.016
PRA-A	38 NS	0.49 ± 0.04	0.35 ± 0.06	0.03 ± 0.00	0.64 ± 0.05	0.73 ± 0.06	10.26 ± 0.19	39.43 ± 0.99	42.30 ± 2.47	0.003 ± 0.000	0.116 ± 0.004
PRA-B	7	1.18 ± 0.04	3.08 ± 0.24	0.45 ± 0.14	7.30 ± 0.17	0.03 ± 0.02	6.02 ± 0.12	27.43 ± 2.67	58.08 ± 2.63	0.006 ± 0.001	0.006 ± 0.000
PRA-B	18	0.95 ± 0.01	0.38 ± 0.03	0.04 ± 0.00	1.92 ± 0.22	0.02 ± 0.02	5.43 ± 0.41	19.25 ± 1.86	21.34 ± 7.38	0.007 ± 0.001	0.008 ± 0.002
PRA-B	29	0.83 ± 0.09	0.22 ± 0.06	0.03 ± 0.00	1.12 ± 0.32	0.04 ± 0.02	5.19 ± 1.54	29.01 ± 10.45	37.16 ± 12.16	0.003 ± 0.001	0.015 ± 0.003
PRA-B	38 S	0.49 ± 0.04	0.35 ± 0.06	0.03 ± 0.00	0.64 ± 0.05	0.73 ± 0.06	10.26 ± 0.19	39.43 ± 0.99	30.91 ± 1.97	0.003 ± 0.000	0.116 ± 0.004
PRA-B	38 NS	0.40 ± 0.03	0.25 ± 0.04	0.03 ± 0.00	0.60 ± 0.07	0.32 ± 0.13	9.07 ± 0.96	30.76 ± 1.87	48.55 ± 1.83	0.002 ± 0.000	0.118 ± 0.008
PRA-D	7	0.83 ± 0.03	3.26 ± 0.18	0.25 ± 0.02	7.50 ± 1.00	0.03 ± 0.01	9.23 ± 0.22	31.61 ± 1.22	65.78 ± 0.27	0.006 ± 0.000	0.006 ± 0.000
PRA-D	18	0.55 ± 0.01	0.20 ± 0.02	0.03 ± 0.00	1.69 ± 0.32	0.00 ± 0.00	6.10 ± 0.42	17.60 ± 0.88	26.83 ± 1.13	0.006 ± 0.000	0.006 ± 0.000
PRA-D	29	0.41 ± 0.02	0.13 ± 0.01	0.03 ± 0.00	0.79 ± 0.07	0.04 ± 0.00	5.11 ± 0.17	23.07 ± 0.89	29.99 ± 1.11	0.002 ± 0.000	0.006 ± 0.000
PRA-D	38 S	0.40 ± 0.03	0.25 ± 0.04	0.03 ± 0.00	0.60 ± 0.07	0.32 ± 0.13	9.07 ± 0.96	30.76 ± 1.87	31.60 ± 1.57	0.002 ± 0.000	0.118 ± 0.008
PRA-D	38 NS	0.55 ± 0.01	0.16 ± 0.03	0.03 ± 0.00	0.65 ± 0.09	0.54 ± 0.22	4.70 ± 0.35	23.81 ± 1.62	27.55 ± 0.35	0.004 ± 0.001	0.124 ± 0.012
