# Supplementary material for: Advances in understanding Norway spruce natural resistance to needle bladder rust infection: transcriptional and secondary metabolites profiling
Source: BMC Genomics. 2022 Jun 13;23:435. doi: 10.1186/s12864-022-08661-y (PMC9190139; doi:10.1186/s12864-022-08661-y)
Supplement: Supplementary file 26 — Additional file 26: Command S1. STAR command. [file 12864_2022_8661_MOESM26_ESM.doc]

**Additional file 26: Command S1. STAR command**

The detailed STAR settings used for mapping and genome index generation.

STAR --runMode genomeGenerate --runThreadN 12 --genomeDir star_index2/ --genomeFastaFiles Pabies01-genome-collapsed3.fa ../ERCC/ERCC92.fa ../SIRV/SIRV_170504a.fasta --sjdbGTFfile Pabies01b_ERCC_SIRV.gtf --sjdbOverhang 100 --genomeSAindexNbases 10 --genomeSAsparseD 5 --limitGenomeGenerateRAM 99000000000
