# Supplementary material for: Advances in understanding Norway spruce natural resistance to needle bladder rust infection: transcriptional and secondary metabolites profiling
Source: BMC Genomics. 2022 Jun 13;23:435. doi: 10.1186/s12864-022-08661-y (PMC9190139; doi:10.1186/s12864-022-08661-y)
Supplement: Supplementary file 29 — Additional file 29: Table S16. RT-qPCR assay details. [file 12864_2022_8661_MOESM29_ESM.docx]

**Additional file 29: Table S16.** **RT-qPCR assay details**

Primer sequences of RT-qPCR assays.

| **Gene ID*** | **Name*** | **Primer sequence (5‘ – 3‘)** | **Amplicon size (bp)** | ***E*** | **R^2^** | **Reference** |
| --- | --- | --- | --- | --- | --- | --- |
| MA_101621g0020 | enhanced disease susceptibility 1 protein (lipase-like PAD4) | F: AAGTTGATACGGAATCCTCTTAC  R: TTCAATTTCTCCTGCTACTTGA | 101 | 0.999 | 0.997 | Trujillo-Moya et al. 2020 |
| MA_10194g0020 | mitogen-activated protein kinase kinase 9 | F: CCTGATGGGTATGGTGGAAAG  R: GCGCACACTCCAGTAAAGATA | 79 | 1.055 | 0.999 | Trujillo-Moya et al. 2020 |
| MA_10313114g0010 | basic endochitinase | F: CCACGGACGAGGACCTATT  R: CGGGTTGTTTATGAGATCGTATCC | 88 | 0.971 | 0.990 | Trujillo-Moya et al. 2020 |
| MA_10427514g0010 | class IV chitinase | F: GAAGAGCTACCATGGACGTG  R: GTTGTTCAGCCCGTCGAA | 94 | 1 | 0.990 | This study |
| MA_10427661g0030 | actin | F: TGAGCTCCCTGATGGGCAGGTGA  R: TGGATACCAGCAGCTTCCATCCCAAT | 104 | 0.9 | 0.998 | Yakovlev et al. 2006 |
| MA_10429865g0020 | disease resistance RPP13 4 | F: CAAATTGGAAGAGCTGGTCATAG  R: CATTGCATCCTCGTGGAAAG | 73 | 0.92 | 0.983 | This study |
| MA_10430127g0010 | salt stress / antifungal | F: AAGGCAGTGAGTTGGAGAG  R: TTTGTGCAGTTGTCAATGGAT | 91 | 0.97 | 0.991 | This study |
| MA_10430573g0010 | flavonoid 3', 5'-hydroxylases | F: GACATGGTCTGGGCAG  R: GTTTGAGAATATTCTGGAGCATG | 145 | 0.91 | 0.990 | Hammerbacher et al. 2018 |
| MA_10431324g0020 | calcium-binding protein CML (calcium-binding CML42-like) | F: TCATACTTGGGACCTCTCTC  R: CAGTAACCCTTGTACGTCTTC | 88 | 0.999 | 0.993 | Trujillo-Moya et al. 2020 |
| MA_10432110g0010 | cinnamyl-alcohol dehydrogenase (sinapyl alcohol) | F: GGATGGTTCTCCCACCTTT  R: CATAGCAATGGTGCTGCTG | 111 | 0.93 | 0.992 | This study |
| MA_10434494g0010 | A Chain Structure Of A Three-Domain Sesquiterpene Synthase: A Prospective Target For Advanced Biofuels Production | F: GGCGTGAATTGCTTAGCTTT  R: ACATACCCAGCTGCCAAC | 91 | 1 | 0.981 | This study |
| MA_10435905g0020 | transcription factor MYC2 | F: TGGCCAGAGGAAACTTTGAG  R: CAGAGTTCAGGGACTGGATTTC | 100 | 0.856 | 0.991 | Trujillo-Moya et al. 2020 |
| MA_10435932g0010 | carotenoid 9,10(9 ,10 )-cleavage dioxygenase 1 | F: CCTGGAAGAGGATGATGGTTAC  R: GCATCAAACACATTGACCTCTG | 81 | 0.94 | 0.997 | This study |
| MA_10437020g0010 | mitogen-activated protein kinase 6 (mitogen-activated kinase homolog MMK1) | F: TCCCTCCGCTATTTCCTATT  R: GCAACTTGCTCATTGGTTTC | 82 | 1 | 0.988 | Trujillo-Moya et al. 2020 |
| MA_1489g0010 | defensin 4 | F: GTTTGCCGAACTGAAGGATTTC  R: GGCTTGTAGCAGTAGCACTT | 77 | 0.91 | 0.999 | This study |
| MA_156769g0010 | probable disease resistance At4g33300 | F: GATGACTCTACTCGCGAAATGT  R: CCATCTCACACCAATCCTTCTC | 109 | 0.94 | 0.999 | This study |
| MA_175884g0010 | farnesyl diphosphate synthase (farnesyl pyrophosphate synthase 1-like) | F: GATCCAGAAGTGATTGGGAAGAT  R: CGTTGAAGTTGGCTCTCATTTG | 104 | 0.94 | 0.995 | This study |
| MA_3160g0010 | toll-interleukin-resistance (TIR) domain | F: TGGAGGAATGCACTTTCGG  R: GCACCAATTGATGGGCAAG | 94 | 0.99 | 0.998 | This study |
| MA_4711g0010 | flavonol synthase | F: GCCAAGTACGTACCCAACAC  R: CACCAACGTCCTGTGCAATA | 99 | 0.99 | 0.999 | This study |
| MA_52889g0010 | calmodulin | F: AAATTGCGGAGTTCAGAGAG  R: CCAGCTCCTTGGTTGTTATG | 75 | 0.963 | 0.980 | Trujillo-Moya et al. 2020 |
| MA_53673g0010 | pathogenesis-related protein 1 (pathogenesis-related PRB1-3-like) | F: CGTGATGGCTTGGGTAAAC  R: TCTCCACACCACTTGAGTATAG | 100 | 0.999 | 0.986 | Trujillo-Moya et al. 2020 |
| MA_5735g0010 | chalcone synthase | F: CTGCCCAGACAATTCTTCC  R: GTAAGTCCGACCTCTCTCAA | 67 | 0.999 | 0.990 | Trujillo-Moya et al. 2020 |
| MA_6204656g0010 | leaf rust 10 disease-resistance locus receptor-like protein kinase-like | F: TACCTTGACTACTCCACCTGAA  R: TTGTCTTGCACAGAGATGGG | 84 | 0.9 | 0.973 | This study |
| MA_66201g0010 | peroxidase (peroxidase 12) | F: AGCTCGCGAGTCGGTTA  R: TCGTGTGGCGAATTTCAGG | 85 | 0.9 | 0.979 | This study |
| MA_7544918g0010 | class IV chitinase | F: TCAGAGTTCAAGCACTTGGC  R: CGCCGCTCCGTAATTGTAA | 94 | 0.94 | 0.994 | This study |
| MA_76780g0010 | flavonoid 3'-monooxygenase (flavonoid 3 -hydroxylase) | F: GCAACCCACAGATGATGAA  R: GACTCCTTTAGCGTCCTTTG | 79 | 0.981 | 0.958 | Trujillo-Moya et al. 2020 |
| MA_8884419g0010, MA_10435699g0020 | ubiquitin (ubiquitin-40S ribosomal S27a) | F: GTTGATTTTTGCTGGCAAGC  R: CACCTCTCAGACGAAGTAC | 101 | 0.91 | 0.997 | Schmidt and Gershenzon, 2008 |
| MA_8921185g0010 | basic endochitinase | F: CCGCATCGGGTTCTACAAA  R: GGCCTCTGGTTGTTACAGTC | 81 | 1.079 | 0.992 | Trujillo-Moya et al. 2020 |
| MA_9401581g0010 | ent-copalyl diphosphate synthase ((-)-linalool synthase) | F: GTTTCGAGCTCTTCGACTACAC  R: TCTCTCCCTCTGTCTGAGTATTG | 104 | 1 | 0.995 | This study |
| MA_985876g0010 | dehydration-responsive RD22 | F: GACGCGGAACATGAATAATACC  R: CAAACTCTATCATGCCCTCAATC | 101 | 0.95 | 1 | This study |
| MA_99372g0010 | cytochrome P450 CYP736A12-like | F: AACGAGGCTCATAGTGAACG  R: TAAACCTCTCCGGCTTGAAC | 89 | 0.95 | 0.998 | This study |
| N/A | flavanone-3-hydroxylase | F: GCAGAGCGTGCACAG  R: GTGAGTTGAGTTCTGTGGAG | N/A | 0.9 | 0.997 | Hammerbacher et al. 2019 |

*ConGenIE database (<http://congenie.org/>); F and R: forward and reverse primer; *E*: amplification efficiency; R^2^: correlation coefficient of standard curve; For some genes the names have been updated in the congenie database (The new names are shown in brackets).

N/A: No information about this in the paper and reference sequence was not submitted to public.
