# Supplementary material for: Advances in understanding Norway spruce natural resistance to needle bladder rust infection: transcriptional and secondary metabolites profiling
Source: BMC Genomics. 2022 Jun 13;23:435. doi: 10.1186/s12864-022-08661-y (PMC9190139; doi:10.1186/s12864-022-08661-y)
Supplement: Supplementary file 30 — Additional file 30: Figure S12. Two reference genes (RGs), actin and ubiquitin included for normalization. [file 12864_2022_8661_MOESM30_ESM.docx]

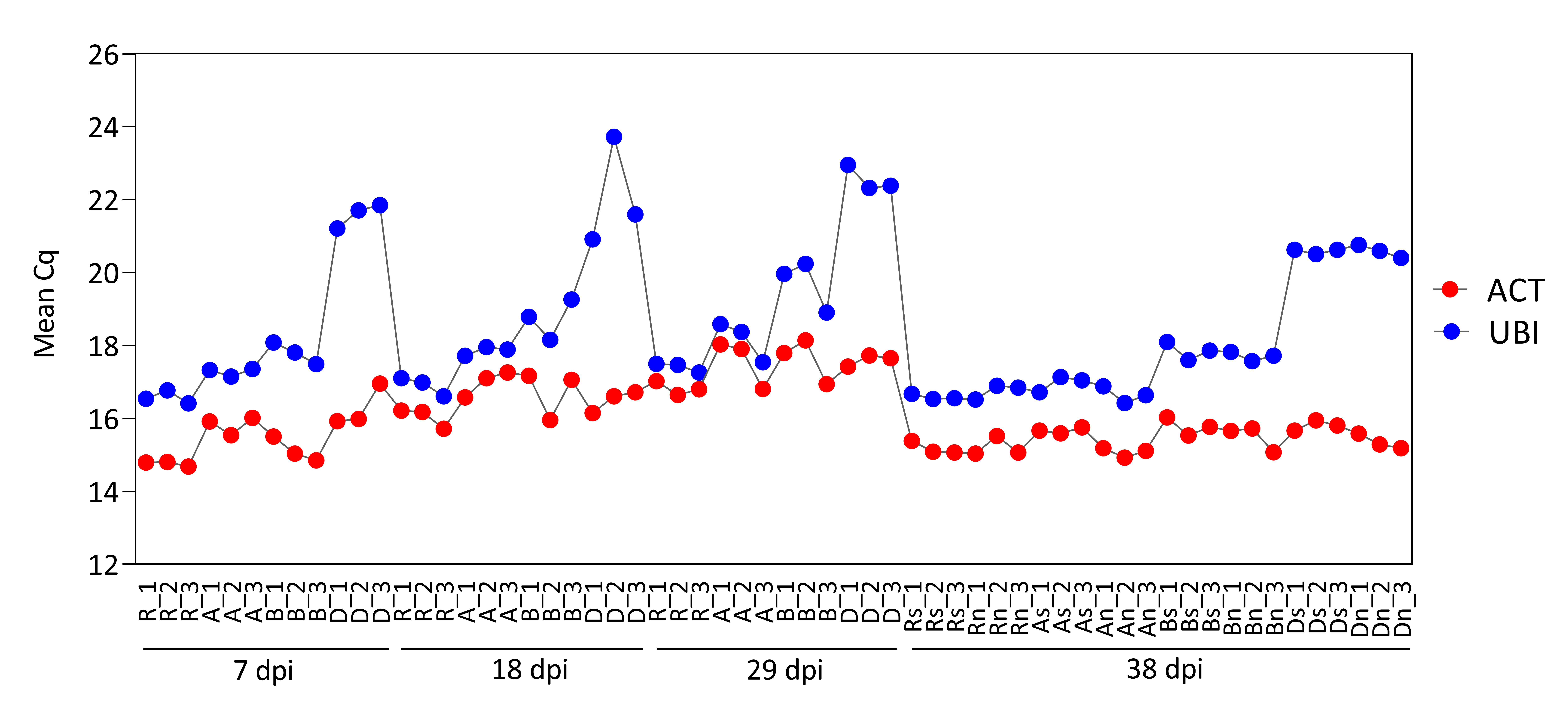


**Additional file 30: Figure S12.** **Two reference genes (RGs), actin and ubiquitin included for normalization**

Both genes were previously found to be suitable RGs for *C. rhododendri* infection studies in *P. abies* (Trujillo-Moya et al. 2020), but ubiquitin was excluded from the analysis due to its unequal expression among the investigated genotypes.
